# An integrated hyperspectral imaging and genome-wide association analysis platform provides spectral and genetic insights into the natural variation in rice

**DOI:** 10.1038/s41598-017-04668-8

**Published:** 2017-06-30

**Authors:** Hui Feng, Zilong Guo, Wanneng Yang, Chenglong Huang, Guoxing Chen, Wei Fang, Xiong Xiong, Hongyu Zhang, Gongwei Wang, Lizhong Xiong, Qian Liu

**Affiliations:** 1 National Key Laboratory of Crop Genetic Improvement and National Center of Plant Gene Research, Huazhong Agricultural University, Wuhan, 430070 China; 2 Agricultural Bioinformatics Key Laboratory of Hubei Province, Huazhong Agricultural University, Wuhan, 430070 China; 3 Britton Chance Center for Biomedical Photonics, Wuhan National Laboratory for Optoelectronics, Huazhong University of Science and Technology, Wuhan, 430074 China; 4 MoE Key Laboratory for Biomedical Photonics, Department of Biomedical Engineering, Huazhong University of Science and Technology, Wuhan, 430074 China

## Abstract

With progress of genetic sequencing technology, plant genomics has experienced rapid development and subsequently triggered the progress of plant phenomics. In this study, a high-throughput hyperspectral imaging system (HHIS) was developed to obtain 1,540 hyperspectral indices at whole-plant level during tillering, heading, and ripening stages. These indices were used to quantify traditional agronomic traits and to explore genetic variation. We performed genome-wide association study (GWAS) of these indices and traditional agronomic traits in a global rice collection of 529 accessions. With the genome-level suggestive *P*-value threshold, 989 loci were identified. Of the 1,540 indices, we detected 502 significant indices (designated as hyper-traits) that exhibited phenotypic and genetic relationship with traditional agronomic traits and had high heritability. Many hyper-trait-associated loci could not be detected using traditional agronomic traits. For example, we identified a candidate gene controlling chlorophyll content (Chl). This gene, which was not identified based on Chl, was significantly associated with a chlorophyll-related hyper-trait in GWAS and was demonstrated to control Chl. Moreover, our study demonstrates that red edge (680–760 *nm*) is vital for rice research for phenotypic and genetic insights. Thus, combination of HHIS and GWAS provides a novel platform for dissection of complex traits and for crop breeding.

## Introduction

With the growing demand to support and accelerate functional genomics and plant breeding for agronomic traits, large numbers of plant parameters need to be accurately quantified^1^. However, most of traditional phenotypic tools are destructive, measure only a limited number of phenotypes, and hinder the development of the study of functional genomics and crop breeding^2^. To address the challenge of high-throughput screening of increasing genetic resources, plant phenomics is rapidly developing, and phenotyping platforms have been developed^3, 4^. In recent years, phenomics has been frequently used to bridge the gap between genotypes and phenotypes and environmental factors^5^, and provides a new approach to expedite proceedings in understanding the environmental responses and identifying gene function of plant^6^.

To meet the food demands of the world’s population, it is critical to improve crop productivity^7^. Rice is an important food crop worldwide and has been adopted as a model plant in plant science research^8^. Biomass, leaf area, and chlorophyll contribute to rice yield; therefore, methods for accurately and non-destructively quantifying these phenotypic traits are urgently needed^9–11^. In addition, these traditional agronomic traits are complex and poorly inherited^12^ and should be dissected into simpler and more heritable traits^13^.

A hyperspectral imaging system that integrates spectroscopic and imaging techniques produces a three-dimensional dataset containing two spatial dimensions and one spectral dimension^14^. Currently, hyperspectral imaging has been widely used as a new technology to non-destructively capture plant phenotypes, including analyzing small-scale sugar beet disease symptoms^15^, identifying the leaf pigment status of *Arabidopsis* mutants^16^ and predicting the above-ground biomass of individual rice plants^17^. With the availability of low-cost genotyping technologies and easy access to the germplasm resources, genome-wide association study (GWAS) are widely used to link genotype and phenotype and to explore allelic variation^7^. In plants, GWAS have been conducted for agronomic traits and metabolites to understand genetic architecture^18–20^. To our knowledge, there are no GWAS of hyperspectral traits at the whole-plant level to reveal natural genetic variation in plants.

In our study, we developed a high-throughput hyperspectral imaging system (HHIS) that is able to obtain thousands of indices to quantify traditional agronomic traits, including biomass-related traits, green leaf area (GLA), and chlorophyll content (Chl), at the whole-plant level. We used this system to explore the genetic variation in rice. With hyperspectral variation, we identified many significant loci associated with indices of potential importance in genetic research and rice breeding. The combination of HHIS and GWAS provides a powerful strategy for understanding natural genetic variation and accelerating crop improvement.

## Results

### High-throughput hyperspectral imaging system (HHIS)

**Figure 1:**
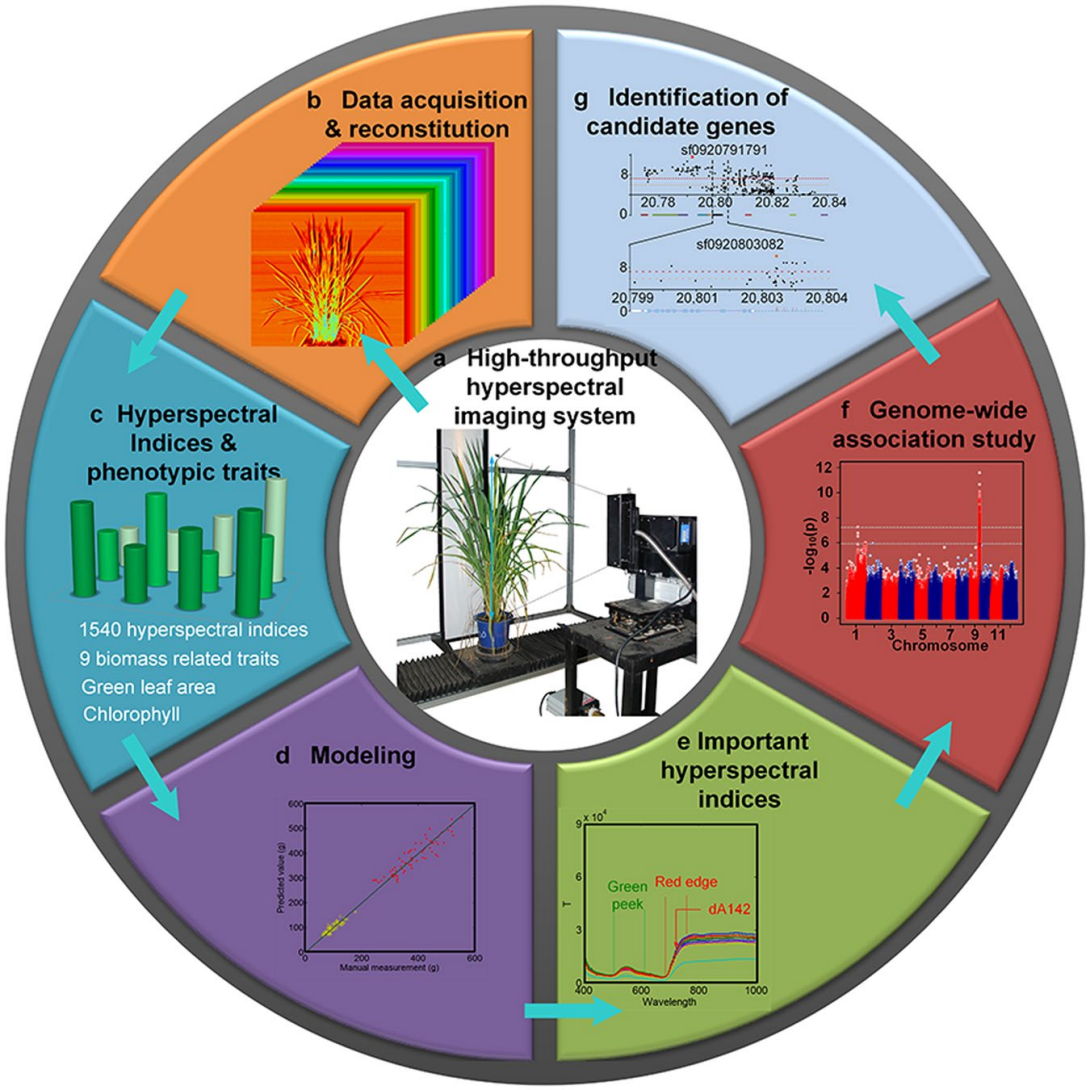
Combination of HHIS and GWAS. (**a**) The HHIS was designed to automatically screen the plant hyperspectral data, and the original data from the HHIS acquisition consisted of a binary data stream. (**b**) A total of 250 images were extracted from the binary data stream via data reorganization, and these images were then used to perform segmentation. (**c**) Hyperspectral indices (including total Reflectance (T), average reflectance (A), first derivative (dT, dA), second derivative (ddT, ddA), and hyperspectral characteristic index (CPT, CPA)) were extracted from the processed images. (**d**) The hyperspectral indices were used to build models of the rice phenotypic traits (including dry weight (DW), green leaf area (GLA), and chlorophyll content (Chl)). (**e**) Important hyperspectral indices can be obtained from the results of these models. (**f**) GWAS of hyperspectral indices. (**g**) Candidate gene identification based on GWAS results.

To non-destructively screen the plant phenotypic traits of rice germplasm resources throughout the growth period, a HHIS, which is composed of a translation stage, a translation stage controller, a halogen lamp, a halogen lamp controller, a hyperspectral camera, a white board, and a workstation, was designed to obtain hyperspectral data of rice plants (Fig. 1a). The overall experimental design using the HHIS is described in Supplementary Fig. 1. The rice samples contained 529 rice accessions mainly from two subspecies (*indica* and *japonica*)^20^. A total of 2,700 GB of data (~799,500 images) were collected for three growth stages (tillering stage, heading stage, and ripening stage; hyperspectral images are presented in Supplementary Fig. 2). After HHIS acquisition, the original binary data stream from one rice plant sample was reorganized to generate hyperspectral images of 250 different wavelengths (Fig. 1b). Then, three image processing steps, including image segmentation, image masking, and data analysis, were used to extract the projected areas (S) and hyperspectral indices of the rice plants (Fig. 1c). The first extracted hyperspectral indices were total reflectance (T), others included the derived hyperspectral indices. Finally, five types of hyperspectral indices, including T, average reflectance (A), first derivative (dT, dA), second derivative (ddT, ddA), and hyperspectral characteristic index (CPT, CPA) (Supplementary Table 1), were extracted from the hyperspectral original data for each rice plant. The hyperspectral indices were then combined with the manual measurement of phenotypic traits of the same rice accessions to generate models for predicting dry weight (DW), GLA, and Chl (the abbreviations of conventional phenotypic traits that are mentioned in this study are presented in Supplementary Table 2), with linear stepwise regression (LSR) (Fig. 1d) and classification for growth periods and subspecies. From the view of modeling and classified results, several key hyperspectral indices were identified (Fig. 1e). Including the time required to transport rice plants to the imaging area, the efficiency of the HHIS was ~8 min per plant.

### Performance evaluation of the HHIS

During the three critical growth and development stages, 11 phenotypic traits were measured manually (the standard deviations, average values, and coefficients of variation are shown in Supplementary Table 3). As mentioned in Supplementary Table 4, a perfect relationship was noted between DW and other biomass-related traits; therefore, DW was used to represent the biomass-related traits.

**Figure 2:**
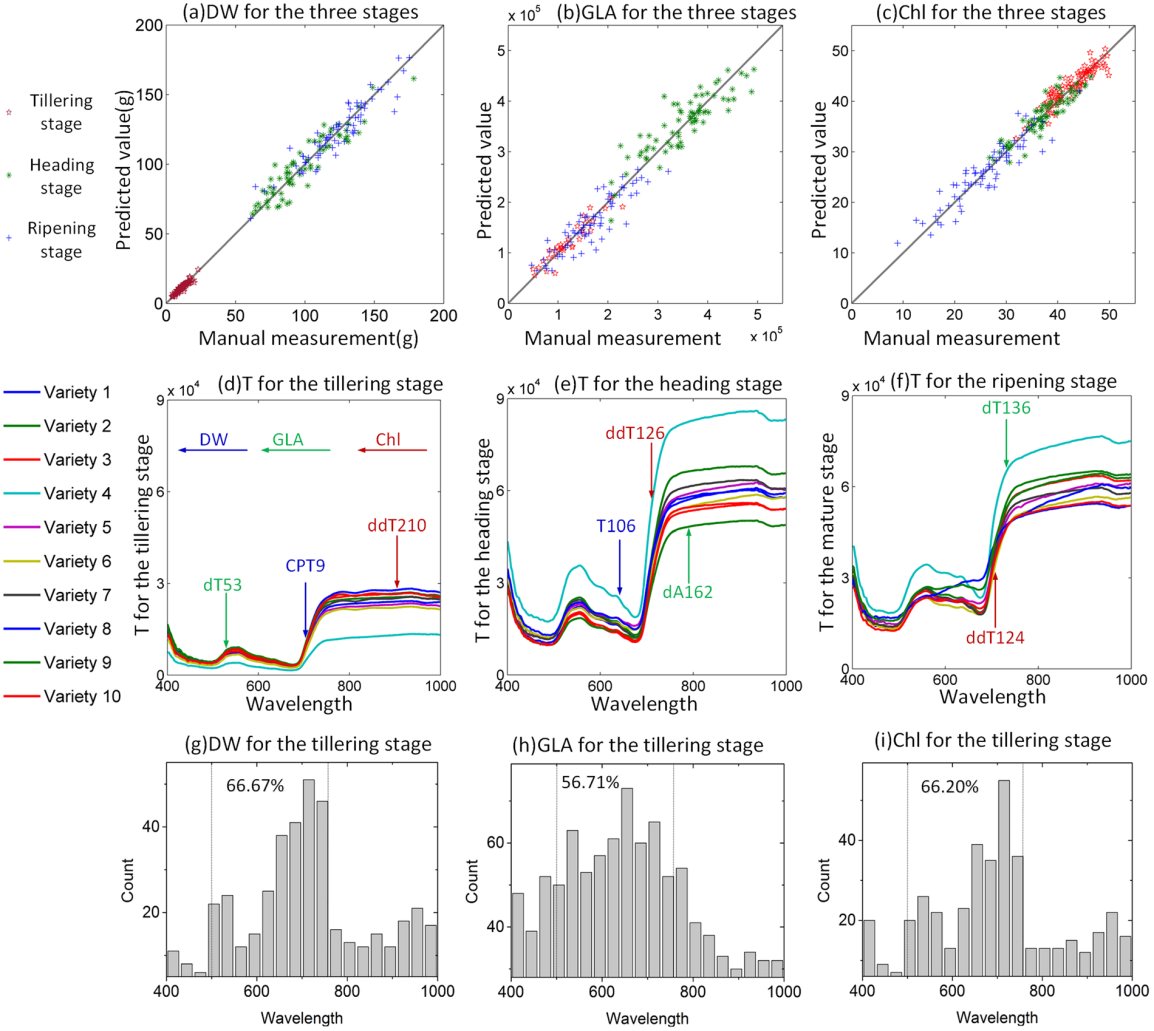
Performance of the LSR for the three growth stages. (**a**) Scatter plot of HHIS measurements versus manual measurements of DW for the three growth stages. (**b**) Scatter plot of HHIS measurements versus manual measurements of GLA for the three growth stages. (**c**) Scatter plot of HHIS measurements versus manual measurements of Chl for the three growth stages. (**d**) Total reflectance for the tillering stage. The blue arrow represents the important hyperspectral indices for DW, the green arrow represents the important hyperspectral indices for GLA, and the red arrow represents the important hyperspectral indices for Chl. (**e**) Total reflectance for the heading stage, with the same arrow coding as described above. (**f**) Total reflectance for the ripening stage, with the same arrow coding as described above. (**g**) The wavelength frequency distribution of the hyperspectral indices that had correlation coefficients with DW greater than 0.3 for the tillering stage. (**h**) The wavelength frequency distribution of the hyperspectral indices that had correlation coefficients with GLA greater than 0.3 for the tillering stage. (**i**) The wavelength frequency distribution of the hyperspectral indices that had correlation coefficients with Chl greater than 0.3 for the tillering stage.

LSR was used to build models between the hyperspectral indices and the phenotypic traits (Supplementary Table 5). Figure 2a–c presents the scatter plots of DW for the three growth stages (Scatter plots of all the phenotypic traits are described in Supplementary Fig. 3). The determination coefficients (R²) and the mean absolute percentage error (MAPE) values of the DW, GLA, and Chl for the three growth stages ranged from 0.734 to 0.877 and 2.97% to 13.36%, respectively. After LSR, the rice samples were then randomly divided into 5 groups, and 5-fold cross-validation was used to test these models (Supplementary Table 6). For all the validation sets, the R² and MAPE values of the DW, GLA, and Chl ranged from 0.722 to 0.856 and 3.17% to 15.47%, respectively. Supplementary Figs 4, 5, 6, 7, 8, 9, 10, 11 and 12 showed the results of the repeatability test of these models. The results of principal component analysis (PCA) and LSR between the phenotypic traits and hyperspectral indices are presented in Supplementary Table 7, and the results of estimating the phenotypic traits with different spectral resolutions are presented in Supplementary Table 8.

The specific models (Supplementary Table 5) demonstrate that the DW was mainly affected by *CPT*_*9*_ (red edge area), *T*_*106*_ (653 *nm*) (blue arrows in Fig. 2d–f), and *S* for the three growth stages. For the GLA, the important hyperspectral indices for the three growth stages were *dT*_*53*_ (525 *nm*), *dA*_*162*_ (788 *nm*), and *dT*_*136*_ (725 *nm*) (green arrows in Fig. 2d–f). For Chl, the main hyperspectral indices were *ddT*_*210*_ (903 *nm*), *ddT*_*126*_ (701* nm*), and *ddT*_*124*_ (696 *nm*) (red arrows in Fig. 2d–f). Supplementary Fig. 13 presents all the important hyperspectral indices of the models for Chl for the three growth stages. Four wavelengths of these hyperspectral indices pointed to an adjacent area. The central wavelength of 696, 701, 708, and 720 *nm* was 712 *nm*. Thus, the area near 712 *nm* was important for predicting Chl of the rice plants. In this study, the vast majority of the red edge positions were at approximately 710 *nm* (Fig. 2d–f). Curran *et al*. demonstrated that the red edge can be used to estimate leaf Chl^21^. Moreover, our supplementary study demonstrated that the red edge could also be used to estimate Chl at the leaf level of individual rice plant^22^. All these similar results indicate that the red edge is very important to plant Chl at the plant or leaf level. According to Fig. 2d–f, the important hyperspectral indices were mainly noted in the obvious change zone of the curve. Figure 2g–i presents the wavelength frequency distributions of the hyperspectral indices for which the correlation coefficients with DW, GLA, and Chl were greater than 0.3 for the tillering stage. The results of all the phenotypic traits for the three growth stages are presented in Supplementary Figs 14, 15 and 16. The corresponding regions shown in Fig. 2d–i were similar, indicating that the region from 500 to 760 *nm* was important to these phenotypic traits.

The uniformization of all the original hyperspectral indices of nondestructive samples for the three growth stages is presented in Supplementary Fig. 17. The average reflectance values for the three growth stages of ten varieties, which were randomly selected from all the samples, are presented in Supplementary Fig. 18. The differences between these ten varieties were small at the tillering stage, followed by the heading and ripening stages. Moreover, the differences were mainly manifested in the region from 500 to 760 *nm*. This result was similar to that noted in the above paragraph.

The important wavelengths for classifying the growth stages and the subspecies are presented in Supplementary Figs 19 and 21, respectively. The frequency distribution was also primarily focused in the region from 500 to 760 *nm*. This region corresponded to the results illustrated in Supplementary Fig. 18. The important hyperspectral indices were then used to perform stepwise discriminant analysis for the growth stages and subspecies. The classified results are presented in Supplementary Figs 20 and 22. The correct rates of grouping for the cross validation sets were 97.3% and 93.2%, respectively.

### Hyperspectral variation in a natural population

We determined the levels of 1,540 hyperspectral indices at the whole-plant level for three critical growth and development stages. Abundant hyperspectral variation was observed in a diverse global collection containing 529 rice accessions (Supplementary Table 9). These indices could distinguish different stages and reflect the population structure (Supplementary Figs 19, 20, 21 and 22). We also phenotyped the population for traditional agronomic traits (Chl for the three growth stages mentioned above and dry/fresh panicle weight (D/FPW) at the ripening stage). Bivariate REML (restricted maximum likelihood) analyses based on single-nucleotide polymorphisms (SNPs) were performed to estimate genetic correlation coefficients and heritability^23^. With r_p_ (Pearson’s correlation coefficient with the agronomic traits) ≥0.5, r_g_ (genetic correlation coefficient with the agronomic traits) ≥0.5, and *h* ^*2*^ (genetic variances proportional to the total variances) ≥0.5, we identified 502 indices of potential significance (designated as ‘hyper-traits’ hereafter), which has potential in breeding selection at earlier stages in a high-throughput, nondestructive, and dynamic manner (Supplementary Table 10). For example, the index *ddT*_*124*_ (ddT at the wavelength 696 *nm* at the heading stage; r_p_ = 0.68, r_g_ = 0.67 with dry panicle weight (DPW); *h* ^*2*^ = 0.75) could be used as a reliable indicator to select rice material with high yield potential at the heading stage. Further, these 502 hyper-traits were enriched at wavelengths from 680 to 720 *nm*, which is exclusively the red edge in spectroscopy, indicating the significance of the red edge in rice research (Supplementary Fig. 23).

### GWAS with the HHIS

**Figure 3:**
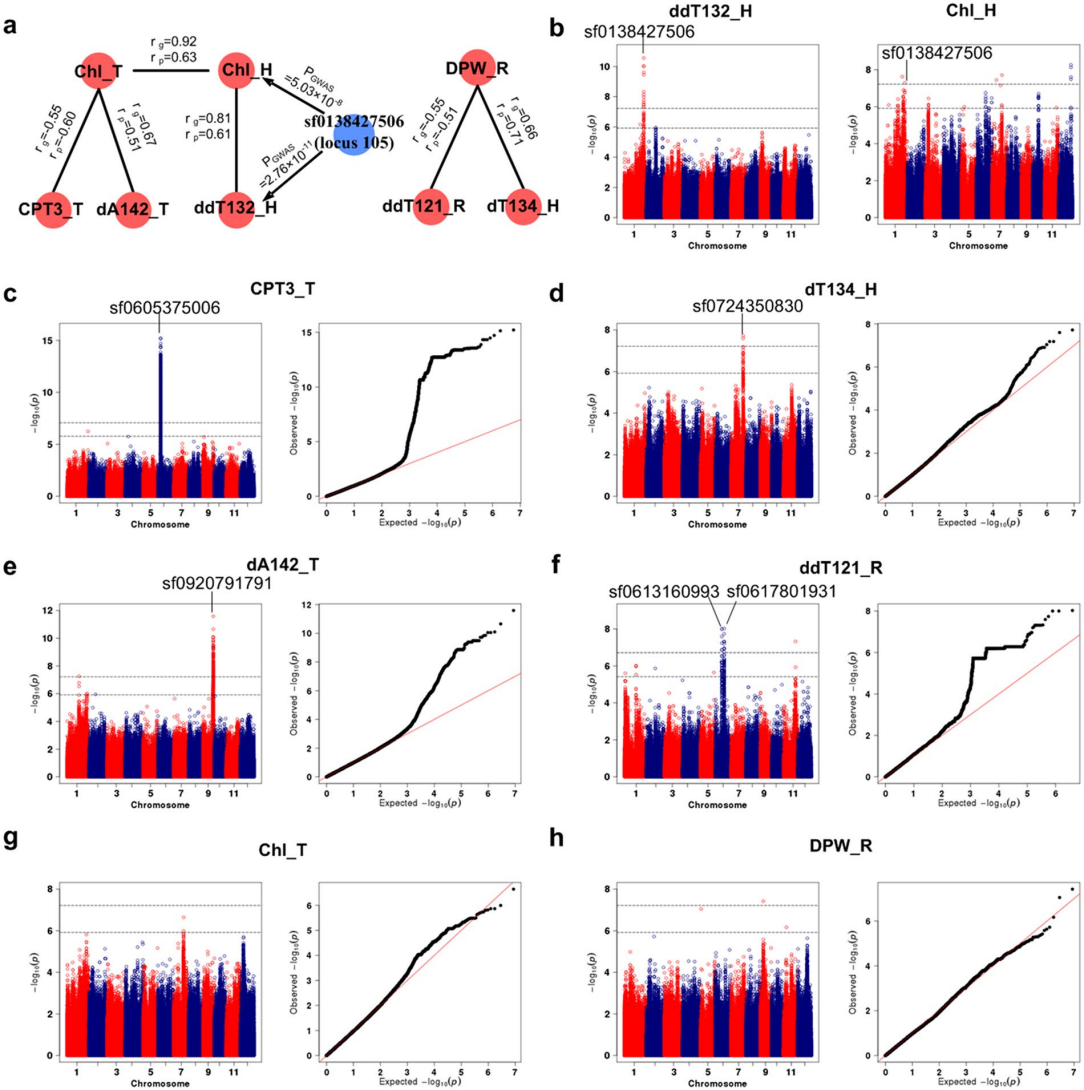
GWAS of hyperspectral indices and agronomic traits. (**a**) Networks reflecting the relationships between indices and agronomic traits based on genetic and phenotypic correlation. (**b**) Manhattan plots of index *ddT*_*132*_ *_H* and Chl_H. (**c**–**h**) Manhattan plots (left) and quantile-quantile plots (right) of indices *CPT*_*3*_ *_T* (**c**), *dT*_*134*_ *_H* (**d**), *dA*_*142*_ *_T* (**e**), and *ddT*_*131*_ *_R* (**f**) and agronomic traits Chl_T (**g**) and DPW_R (**h**). ‘_T’, ‘_H’, and ‘_R’ indicate the tillering, heading, and ripening stages, respectively. For Manhattan plots, −log10 *P*-values from a genome-wide scan are plotted against the positions of the SNPs on each of 12 chromosomes, and the higher/lower horizontal gray dashed lines indicate the genome-wide significant/suggestive *P*-value thresholds. For quantile-quantile plots, the horizontal axis shows −log10-transformed expected *P*-values, and the vertical axis indicates −log10-transformed observed *P*-values. The lead SNPs of associated loci with strong signals are presented.

To explore allelic variation associated with these hyperspectral indices, we performed GWAS for all the indices and phenotypic traits. After Bonferroni correction for multiple tests, we scanned 9,379 lead SNPs exceeding the suggestive genome-wide *P*-value threshold. Considering linkage disequilibrium (LD) in rice, we defined a chromosomal region in which the distance of adjacent pairs of SNPs was less than 300 *kb* as a locus^20, 24, 25^. As a result, 989 loci and 23,264 associations were scanned in our study (Supplementary Table 11). In total, 743, 828, and 840 loci were detected with the indices at the tillering, heading, and ripening stages, respectively. In total, 540 loci were identified for all three growth stages, suggesting that these loci are indispensable in all whole growth and development stages and that many other loci function in stage-specific patterns. Of the 989 loci, 102, 268, 787, 758, 780, 822, 248, and 258 loci were identified by index types T, A, dA, dT, ddA, ddT, CPA, and CPT, respectively. The average numbers of detected loci per index for the eight types were 0.4, 1.1, 3.1, 3.0, 3.1, 3.3, 12.4, and 12.9, indicating that hyperspectral characteristic indices (CPA and CPT) and derivative indices (dA, dT, ddA, ddT) outperformed raw indices (T and A) in the GWAS. In our study, association signals of many loci were strong, indicating that GWAS of hyperspectral indices is a promising strategy to explore allelic variation. For the index *CPT*_*3*_ at the tillering stage, for example, the *P*-value of the lead SNP sf0605375006 was 6.05 × 10^−16^ in the *indica* subpopulation; this SNP was in LD (r^2^ > 0.25) with 3,498 SNPs with *P*-values < 10^−4^. For the index *dA*_*233*_ at the heading stage, the *P*-value of the lead SNP sf0719864659 was 1.17 × 10^−11^ in the whole population; this characteristic was in LD with 633 SNPs with *P*-values < 10^−4^. For the index *ddA*_*137*_ at the ripening stage, the *P*-value of the lead SNP sf0820477603 was 2.39 × 10^−10^ in the whole population; this SNP was in LD with 603 SNPs with *P*-values < 10^−4^. A total of 55 loci were associated with traditional agronomic traits, of which 54 were also identified by indices (Supplementary Table 12). Among the 54 loci, the lead SNP sf0138427506 was associated with both Chl (*P*_GWAS_ = 5.03 × 10^−8^) and the hyper trait *ddT*_*132*_ (*P*_GWAS_ = 2.76 × 10^−11^; r_g_ = 0.81, r_p_ = 0.61, *h* ^*2*^ = 0.60) at the heading stage (Fig. 3a–b). Further, the wavelengths of hyperspectral indices co-localized with the phenotypic traits were also enriched from 680 to 760 *nm*, indicating the usefulness of red edge-related indices in understanding the genetic bases of important phenotypic traits (Supplementary Fig. 24).

In addition, we identified some loci not detected with traditional traits that were significantly associated with the hyper-traits. The lead SNPs sf0605375006 (in locus 487) and sf0920791791 (in locus 766) failed to be detected with Chl at the tillering stage but were associated with *CPT*_*3*_ (*P*_GWAS_ = 6.05 × 10^−16^, r_g_ = −0.55, r_p_ = −0.60, *h* ^*2*^ = 0.68) and *dA*_*142*_ at the tillering stage (*P*_GWAS_ = 2.59 × 10^−12^, r_g_ = 0.67, r_p_ = 0.51, *h* ^*2*^ = 0.77), respectively (Fig. 3a,c,e,g). The lead SNPs sf0617801931 (in locus 518) and sf0724350830 (in locus 618) failed to be detected with DPW but were associated with *ddT*_*121*_ at the ripening stage (*P*_GWAS_ = 9.43 × 10^−9^, r_g_ = 0.55, r_p_ = 0.51, *h* ^*2*^ = 0.55) and *dT*_*134*_ at the heading stage (*P*_GWAS_ = 1.92 × 10^−8^, r_g_ = 0.66, r_p_ = 0.71, *h* ^*2*^ = 0.62), respectively (Fig. 3a,d,f,h). These results indicate that the hyperspectral indices, especially the hyper-traits, are valuable for revealing the genetic variation harbored in rice and for understanding the genetic bases of complex traits^25^. The identification of the underlying genes of these markedly significant loci warrants further investigation.

### Candidate gene identification

**Figure 4:**
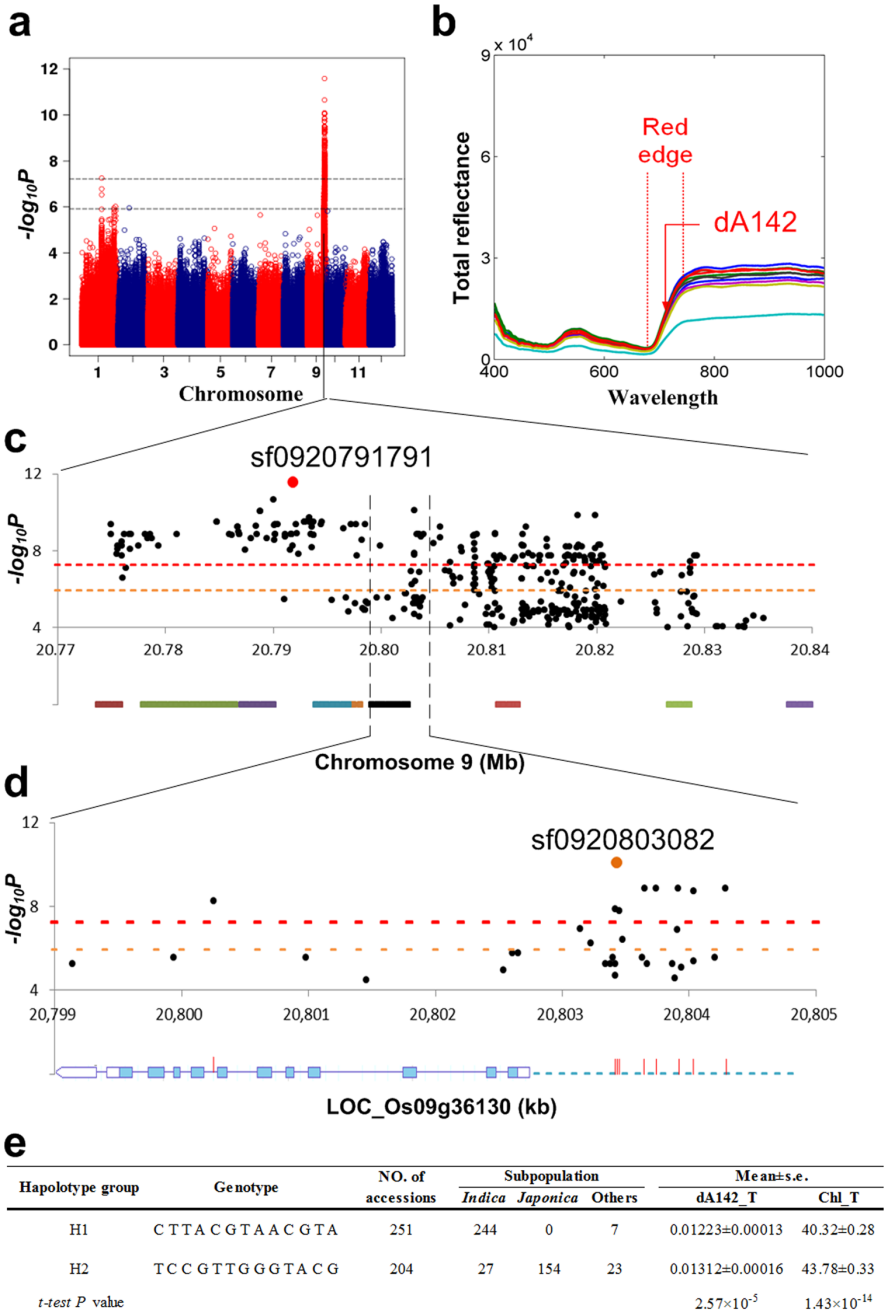
Candidate gene identification of loci associated with the red edge-related index *dA*_*142*_ (genetically and phenotypically correlated with Chl) at the tillering stage. (**a**) Manhattan plot of index *dA*_*142*_. (**b**) Total reflectance values ranging from 400 to 1,000 *nm* of 10 accessions at the tillering stage. (**c**) The 70-*kb* region around the lead SNP sf0920791791. The red/orange horizontal dashed lines indicate the significant/suggestive *P*-value thresholds. The bars with different colors at the bottom represent the genes in the genomic region. (**d**) The 6-*kb* region containing the gene LOC_Os09g36130. Regarding the gene structure at the bottom, the red lines, blue dashed lines, purple lines, white box, and blue box indicate significant SNPs, a promoter region, an intron, an untranslated region (UTR), and an exon, respectively. (**e**) Haplotype analysis of the candidate gene.

Locus 766, associated with *dA*_*142*_ at the tillering stage mentioned above, was detected with 28 indices at the tillering stage, of which 24 were related to the red edge, such as *CPT*_*14*_ (the red edge area of average reflectance) (Fig. 4a–b and Supplementary Table 11). Based on the results mentioned above, we proposed that the function of the causal gene should be related to chlorophyll. In this locus, a gene, LOC_Os09g36130, encoding an unknown protein approximately 7 *kb* away from lead SNP sf0920791791 is predicted to be located in the chloroplast and is expressed mainly in the leaf blade, especially at the tillering stage, based on public databases (Fig. 4c and Online Methods). The gene has no paralogs in rice and only one orthologous gene (AT2G21385, CGLD11 (**C**onserved in the **G**reen **L**ineage and **D**iatoms **11**)) in *Arabidopsis*, which is also predicted to function in chloroplasts. Forty-two chloroplast-related genes are positively co-expressed with the candidate gene (Pearson correlation coefficients > 0.7) (Supplementary Table 13). Among the co-expressed genes, *SGRL* (Stay-Green Like, LOC_Os04g59610) is mainly expressed in green tissues and is involved in chlorophyll degradation^26^. These results indicate that the candidate gene maybe the causal gene underling the locus. This gene and its prompter region (2 *kb* upstream of TSS (transcription start site)) contain 13 significant SNPs, of which 11 SNPs are in nearly complete LD (r^2^ > 0.93) with the lead SNP (Fig. 4d). These SNPs are located in the promoter region, except for one SNP in the intron, and the most significant SNP, sf0920803082 (*P* = 8.04 × 10^−11^, r^2^_LD_ with the lead SNP = 0.97), is located 630 *bp* (base pair) upstream of the TSS. These results suggest that the natural variation in the promoter region of this gene may result in the expression polymorphism, ultimately contributing to phenotypic diversity. Furthermore, we performed haplotype analysis of this gene based on these significant SNPs (Fig. 4e). The accessions in our study were mainly classified into two haplotype groups: H1 (n = 251) and H2 (n = 204). The genotypes of the most significant SNP sf0920803082 of this gene were C and T for H1 and H2, respectively. The levels of *dA*_*142*_ and Chl at the tillering stage of H2 were significantly increased compared with those of H1 (t-test, *P* = 2.57 × 10^−5^ and 1.43 × 10^−14^, respectively). These results further indicate that LOC_Os09g36130 could be the causal gene affecting Chl. Furthermore, 97.2% from H1 accessions and 75.5% from H2 (favorable haplotype) accessions belonged to *indica* and *japonica* subspecies, respectively. The Chl of *japonica* subspecies was significantly higher than *indica* at the tillering stage (*P* = 1.23 × 10^−19^, *t-test*). These results indicated that the gene may contribute to the divergence of chlorophyll content between the two main rice subspecies.

## Discussion

To predict biomass, GLA, and Chl with high accuracy and identify related vital spectral bands, the huge of hyperspectral information was analyzed compared with the phenotypic traits in this study. For the specific model, *S* (projected area) was not among the dependent variables at the tillering and heading stages (the correlation between the GLA and Chl and *S* are presented in Supplementary Table 14). This finding indicated that hyperspectral indices were more significant in predicting rice phenotypic traits than RGB image features. Most of the hyperspectral indices that were selected by the specific models were first derivative, second derivative, and other hyperspectral characteristic indices because derivation could obtain more detailed information than in the original data. The differences between these biomass-related traits, GLA, and Chl were embodied in the obvious changes of reflectance. Combined with the modeling, correlation coefficient and classified results, the important hyperspectral indices of the models and the wavelength frequencies are located in the same essential region. We can infer that the region from 500 to 760 *nm* was important for the plant growth. The same conclusion can also be obtained from Supplementary Figs 19, 20, 21 and 22. The important hyperspectral indices and wavelength regions selected by this study can provide a referenced screening tool for future work on huge hyperspectral dimension reduction.

In our study, we performed GWAS of a large number of hyperspectral indices at the whole-plant level for the three critical growth and development stages in a natural rice population. Based on our knowledge, this related study describing natural variation of hyperspectral indices at the whole-plant level in plants has not been reported. Many indices have phenotypic and genetic relationships with important agronomic traits and have high heritability; thus, these indices can be used to select elite rice material in a high-throughput, nondestructive, dynamic and stable selection process at earlier growth and development stages. These indices generally outperform traditional traits in GWAS. Some loci associated with these indices with strong association signals failed to be detected with traditional traits. Thus, these indices can be used to dissect complex agronomic traits. Based on spatio-temporal expression profiles, co-expression, homologous analysis, LD and haplotype analysis, the genes underlying these loci could be identified. GWAS of large-scale hyperspectral indices could provide a novel approach for understanding the genetic bases of complex traits and accelerating crop improvement.

Hyperspectral imaging in our study obtained the indices of the wavelengths ranging from 400 to 1,000 *nm* (including the red edge) with high resolution. Based on the phenotypic modeling and genetic analyses, we discovered that the red edge is quite valuable in rice research, as it can evaluate biomass, GLA, Chl, and yield component from phenotypic and genetic insights, and the indices related to red edge perform well in genetic analyses. The red edge is beyond the visible range; therefore, traditional phenotyping technologies cannot obtain this valuable information. In addition, comparison of the eight different index types revealed that the derivative index types (dT, dA, ddT, ddA, CPT, CPA) could detect more genetic loci than the raw index types (T and A) in the GWAS, which implies that derivative data are necessary when the raw data consist of time or wavelength series.

In addition, we compared the Chl of this study with that of our previous field research (the SPAD value of rice in field, denoted as Field-Chl-2012 and Field-Chl-2013)^27^. The correlation coefficients between the Chl and Field-Chl-2012 at the tillering and heading stage were 0.571 and 0.619, respectively. And the correlation coefficients between the Chl and Field-Chl-2013 at the tillering and heading stage were 0.569 and 0.550, respectively. Moreover, five loci were repeatedly detected with Chl in both our present study and the previous field work in GWAS while only seven common loci associated with Chl were detected in 2012 and 2013 in the previous field work (Supplementary Table 11). These results demonstrate that our phenotypic data and GWAS results are reproducible and instructive in the field research and that these common loci are worthy of further studying.

## Materials and Methods

### Plant material

A global collection including 529 *O. sativa* accessions was used in our study^2, 20, 24^, of which 100 accessions were chosen for conventional measurement and modeling. In 2013, seeds were sown in a field, and three healthy 20-day-old seedlings of each accession were transplanted to three pots at Huazhong Agricultural University. Finally, 91, 71, and 69 accessions were used for conventional measurement and modeling at the tillering, heading, and ripening stages, respectively. Moreover, we determined the levels of hyperspectral indices and agronomic traits of 371, 370, and 328 accessions at the three respective stages for genetic analyses (Supplementary Fig. 1).

### Hyperspectral image acquisition and pre-processing

At each harvest time, pot-grown rice samples from the corresponding stages were imaged twice with two angles (0° and 90°) in a darkroom. The tillering, heading, and ripening stages were imaged from July 8 to July 13, August 3 to September 15, and August 23 to September 29, 2013, respectively. The differences in the time of growth stages were based on the diversity of varieties. The reflectance values of the pot-grown rice were obtained using HHIS^17^. The HHIS consisted of a translation stage, a translation stage controller, a halogen lamp, a halogen lamp controller, a hyperspectral camera, a whiteboard, and a workstation (Fig. 1a). The spectral range and resolution of HHIS were 400 to 1,000 *nm* and 2.4 *nm*, respectively. The focal length and the slit width of the hyperspectral camera were 12 *mm* and 25 *μm*, respectively. The number of scanning lines was 900; therefore, the size of the final three-dimensional data was 1,004 × 250 × 900. Because the intensity of the light was uneven under different wavelengths and the CCD of the camera itself had dark current^28^, the camera should be processed with dark current calibration and gain calibration (with the whiteboard) before image acquisition. To further improve the signal-to-noise ratio, every reflectance was an average of three repeated scans. The actual reflectance was calculated using the following equation:

1 $${r}_{j}=\frac{{I}_{j}-{I}_{dark}}{{I}_{ref}-{I}_{dark}}$$

where *r*_*j*_ indicates the reflectance of the *i-th* wavelength, *I*_*j*_ indicates the raw data of the *i-th* wavelength, *I*_*dark*_ indicates the dark current data, and *I*_*ref*_ indicates the whiteboard data.

### Hyperspectral data processing and analyzing

The hyperspectral data that were obtained from the previous step consisted of a binary data stream. Thus, the data were reorganized to extract images^17^. Here, we did not use ENVI (Exelis Visual Information Solutions, USA), which is typically used to process spectral data to extract an image, because using ENVI to process large-scale data is labor-intensive. Instead, we used LabVIEW (National Instruments, USA) software to write a program to implement automatic extraction of images from the binary data stream that would be fully automated without any human intervention. The detailed steps are presented in Supplementary Fig. 25. After the image processing, a series of hyperspectral indices were calculated from the processed images (Supplementary Table 1).

### Manual measurements of plant biomass, green leaf area, and Chl

After the pot-grown rice was imaged with HHIS, a sample was taken to measure Chl with a SPAD-502 (Konica Minolta, Japan). For each tiller, the measurement point was at the center of the second leaf from top to bottom. Each leaf was measured twice, and four healthy tillers were measured from each plant. The final Chl per plant was the average of eight measurements. The sample was then harvested to measure the GLA with a high-throughput leaf scorer^24^. Subsequently, all the plant parts were used to measure the FW and the DW using an electronic balance. The FW was the weight of all the organs of the rice plant except the root and included the FLW, the FSW, and the FPW. The DW included the DLW, the DSW, and the DPW.

### Modeling and validation

In total, 1,541 independent variables were obtained for each stage; therefore, the data needed to be reduced dimensionally. Regression analysis improved the grass biomass estimation compared with the hyperspectral index, such as the normalized difference vegetation index and the red edge^29^. LSR analysis was used to select some effective hyperspectral indices for these phenotypic traits when there were numerous independent variables, and some of them were not completely independent of each other.

After the LSR, 5-fold cross validation was used to evaluate the accuracy of the predicted model. The average R² of the modeling set, the MAPE, and the SD of absolute percentage error (SD_APE_) of the prediction set were calculated to evaluate the performance of the model as follows:

2 $${MAPE}=\frac{1}{n}\times \sum _{i=1}^{n}|({Y}_{i}-{y}_{i})/{Y}_{i}|\times 100 \% $$

3 $$S{D}_{APE}=\sqrt{\frac{1}{n}\sum _{i=1}^{n}{(yi-\bar{y})}^{2}}$$

4 $${y}_{i}={Y}_{i}-\bar{y}$$

5 $$\bar{y}=\frac{1}{n}\sum _{i=1}^{n}({Y}_{i}-{y}_{i})$$

where *n* indicates the number of samples used in the model, *Y*_*i*_ indicates the manual measurement, and *y*_*i*_ indicates the predicted value of the model. This step was programmed and achieved using LabVIEW.

### Genetic analyses

Bivariate REML analysis was performed using the software GCTA to estimate genetic correlation and heritability (defined as the genetic variances proportional to the total variances)^23^. Detailed information of the GWAS method has been described in previous studies^2^. The GWAS involved 4,358,600, 2,863,169, and 1,959,460 SNPs (minor allele frequency ≥0.05 and number of accessions with minor allele ≥6) in the whole, *indica*, and *japonica* populations, respectively. A linear mixed model was adopted for the GWAS using the software FaST-LMM^30^. The effective number of independent SNPs (N) was estimated with the GEC tool^31^. Genome-wide suggestive (1/N) and significant (0.05/N) *P*-value thresholds were set to control the false-positive rates of the GWAS. The suggestive/significant *P*-value thresholds were 1.21 × 10^−6^/6.03 × 10^−8^, 1.66 × 10^−6^/8.30 × 10^−8^, and 3.81 × 10^−6^/1.91 × 10^−7^ for the whole, *indica*, and *japonica* populations, respectively. To determine the independent lead SNPs from multiple significant SNPs associated with a given trait, the function–clump from the tool ‘Plink’ was used to remove the dependent SNPs caused by LD (r^2^ > 0.25)^32^. The LD statistic r^2^ was estimated by the function–ld from Plink. The lead SNPs, which surpassed the suggestive threshold and were in LD (r^2^ > 0.25) with ≥5 SNPs (*P*_GWAS_ < 1 × 10^−4^), remained in our study.

### Candidate gene identification

Based on GWAS results, candidate genes were chosen from the genes around the lead SNPs of associated loci (100 *kb* upstream and downstream). The databases ‘Rice DB’ (http://ricedb.plantenergy.uwa.edu.au/)^33^ and ‘RICECHIP.ORG’ (http://www.ricechip.org/) provided the predicted subcellular localization. The website ‘Rice Oligonucleotide Array Database’ (http://www.ricearray.org/)^34^ supplied the co-expression information. The putative homologies in rice and *Arabidopsis* were available in the database ‘CREP’ (http://crep.ncpgr.cn/crep-cgi/home.pl)^35^. *Arabidopsis* information was collected from the website ‘tair’ (http://www.arabidopsis.org/index.jsp). The gene expression profiles were available in the database ‘RiceXPro’ (http://ricexpro.dna.affrc.go.jp/)^36^.

## Electronic supplementary material

Supplementary Table 9

Supplementary Table 10

Supplementary Table 11

Supplementary Table 12

Supplementary Table 13

Supplementary Table 1-8,14 and Figures
