# Supplementary material for: An integrated hyperspectral imaging and genome-wide association analysis platform provides spectral and genetic insights into the natural variation in rice
Source: Sci Rep. 2017 Jun 30;7:4401. doi: 10.1038/s41598-017-04668-8 (PMC5493659; doi:10.1038/s41598-017-04668-8)
Supplement: Supplementary file 6 — Supplementary Table 1-8,14 and Figures [file 41598_2017_4668_MOESM6_ESM.doc]

**An integrated hyperspectral imaging and** **genome-wide association analysis platform provides spectral and genetic insights into the natural variation in rice**

Hui Feng^1, 2, 3, 4^, Zilong Guo^1^, Wanneng Yang^1, 2^, Chenglong Huang^1, 2^, Guoxing Chen^1^, Wei Fang^3, 4^, Xiong Xiong^3, 4^, Hongyu Zhang^2^, Gongwei Wang^1^, Lizhong Xiong^1^ & Qian Liu^3, 4^

^1^National Key Laboratory of Crop Genetic Improvement and National Center of Plant Gene Research, Huazhong Agricultural University, Wuhan 430070, China.

^2^Agricultural Bioinformatics Key Laboratory of Hubei Province, Huazhong Agricultural University, Wuhan 430070, China.

^3^Britton Chance Center for Biomedical Photonics, Wuhan National Laboratory for Optoelectronics, Huazhong University of Science and Technology,Wuhan 430074, China

^4^MoE Key Laboratory for Biomedical Photonics, Department of Biomedical Engineering, Huazhong University of Science and Technology, Wuhan 430074, China

Hui Feng and Zilong Guo contributed equally to this work.

Co-corresponding authors: Lizhong Xiong (lizhongx@mail.hzau.edu.cn. Fax: +86 27 87287092. Tel: +86 27 87281536) and Qian Liu (qianliu@mail.hust.edu.cn. Fax: +86 27 87792034. Tel: +86 27 87792033)

**This supplementary file contains the following information:**

**Supplementary Figures 1-25**

**Supplementary Tables 1-14 (Supplementary Table 9-13 were big datasets presented as separated files)**

**Supplementary References**

**Supplementary Figure 1-25**

**Supplementary Figure 1 The overall experimental design of the rice** population.

Supplementary Figure 2 The hyperspectral images of the same rice plants for the three growth stages.

Supplementary Figure 3 Scatter plots of manual versus automatic measurements of all the phenotypic traits at tillering stage (a-c), heading stage (d-f), and ripening stage (g-i).

Supplementary Figure 4 The results of the repeatability test of coefficients and constant for the DW modeling at the tillering stage.

Supplementary Figure 5 The results of the repeatability test of coefficients and constant for the GLA modeling at the tillering stage.

Supplementary Figure 6 The results of the repeatability test of coefficients and constant for the Chl modeling at the tillering stage.

Supplementary Figure 7 The results of the repeatability test of coefficients and constant for the DW modeling at the heading stage.

Supplementary Figure 8 The results of the repeatability test of coefficients and constant for the GLA modeling at the heading stage.

Supplementary Figure 9 The results of the repeatability test of coefficients and constant for the Chl modeling at the heading stage.

Supplementary Figure 10 The results of the repeatability test of coefficients and constant for the DW modeling at the ripening stage.

Supplementary Figure 11 The results of the repeatability test of coefficients and constant for the GLA modeling at the ripening stage.

Supplementary Figure 12 The results of the repeatability test of coefficients and constant for the Chl modeling at the ripening stage.

Supplementary Figure 13 Important hyperspectral indices of the models for the Chl for the three growth stages.

Supplementary Figure 14 The wavelength frequency distributions of the hyperspectral indices for which the correlation coefficients with FLW, FSW, and FW were greater than 0.3 for the three growth stages.

Supplementary Figure 15 The wavelength frequency distributions of the hyperspectral indices for which the correlation coefficients with DLW, DSW, and DW were greater than 0.3 for the three growth stages.

Supplementary Figure 16 The wavelength frequency distributions of the hyperspectral indices for which the correlation coefficients with WW, GLA, and Chl were greater than 0.3 for the three growth stages.

Supplementary Figure 17 The uniformization of all the original hyperspectral indices of nondestructive samples for the three growth stages.

**Supplementary Figure 18** The average reflectance for ten varieties at the three growth stages.

Supplementary Figure 19 The important wavelengths for classifying the growth stages.

**Supplementary Figure 20** The classified result for the growth stages with hyperspectral indices that were chosen from Supplementary Fig. 19 using stepwise discriminant analysis.

Supplementary Figure 21 The important wavelengths for classifying the subspecies.

Supplementary Figure 22 The classified result for subspecies with hyperspectral indices that were chosen from Supplementary Fig. 21 using stepwise discriminant analysis.

Supplementary Figure 23 The wavelength distributions of hyper-traits.

Supplementary Figure 24 The wavelength distribution of hyperspectral indices co-localized with agronomic traits.

**Supplementary Figure 25** Hyperspectral data analysis pipeline.

**Supplementary Tables 1-14**

**Supplementary Table 1 Hyperspectral indices used in this study (1≤i≤250).**

Supplementary Table 2 The abbreviation of conventional phenotypic traits in this study*.

Supplementary Table 3 The statistic details of the 11 conventional phenotypic traits.

Supplementary Table 4 The correlation coefficient between these phenotypic traits*.

Supplementary Table 5 The results of models for the conventional phenotypic traits with the linear stepwise regression (LSR).

**Supplementary Table 6** The result of 5-fold cross-validation between the conventional phenotypic traits and hyperspectral indices (randomly grouping once).

**Supplementary Table 7** The result of principal component analysis (PCA) and linear stepwise regression (LSR) between the destructive phenotypic traits and hyperspectral indices*****.

**Supplementary Table 8** The results of estimating the biomass, leaf area, and Chl with different spectral resolutions*.

**Supplementary Table 9** All the phenotyping data including hyperspectral indices and agronomic traits in our study.

**Supplementary Table 10** The heritability and genetic correlation with traditional agronomic traits of hyper-traits.

**Supplementary Table 11** GWAS results of hyperspectral indices and agronomic traits.

**Supplementary Table 12** Co-localized loci between hyperspectral indices and agronomic traits.

**Supplementary Table 13** Forty-two chloroplast-related genes positively co-expressed with LOC_Os09g36130.

**Supplementary Table 14** The correlation coefficient between these phenotypic traits and projected area (S).

**Supplementary References**

**Supplementary Figure**


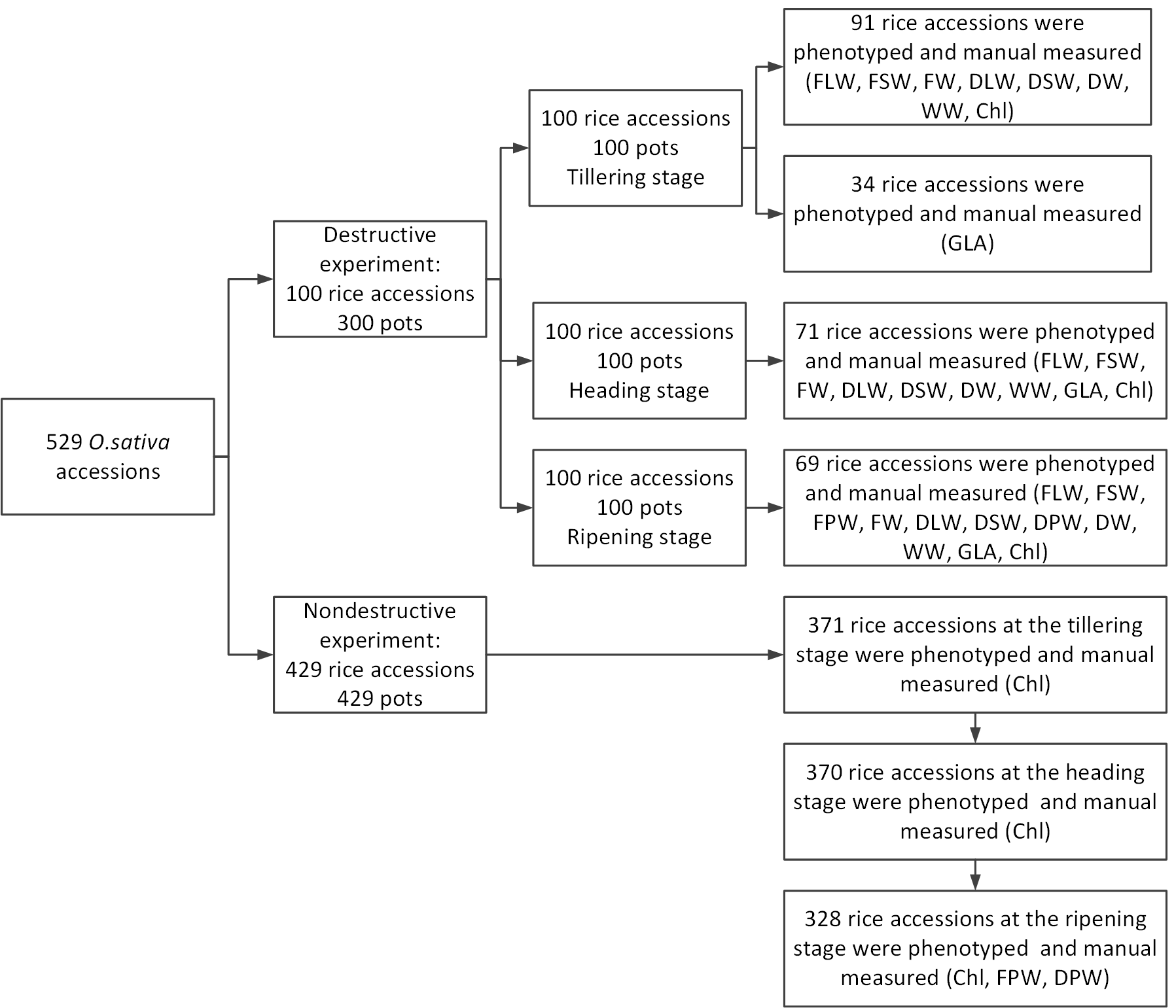


**Supplementary Figure 1 The overall experimental design of the rice population**.

The full name for these phenotypic traits is shown in **Supplementary Table 2**.


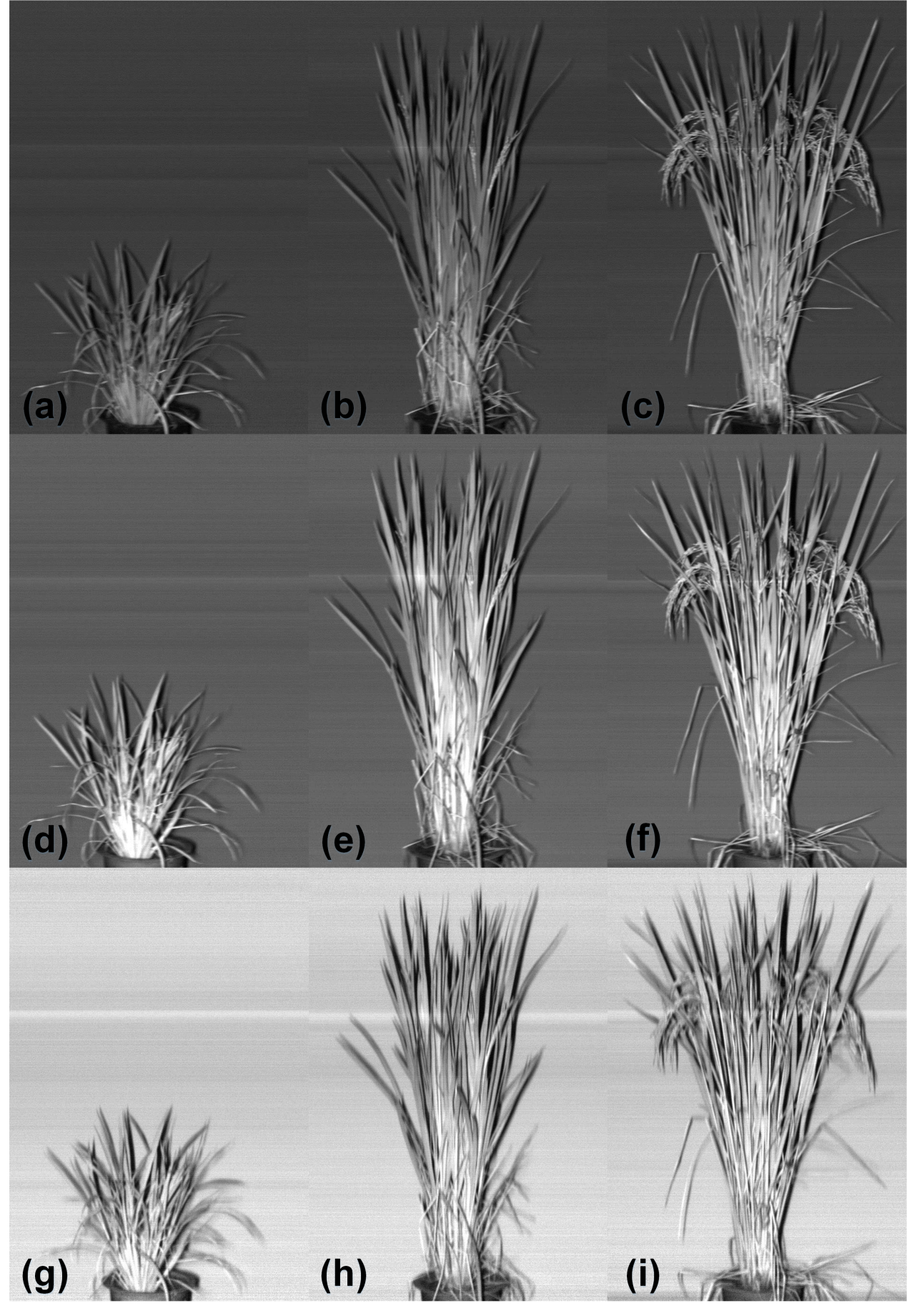


**Supplementary Figure 2 The hyperspectral images of the same rice plants for the three growth stages.** The rice plants a, d, and g: the tillering stage; b, e, and h: the heading stage; c, f, and i: the ripening stage. a, b, and c were the hyperspectral images of 710 *nm*; d, e, and f were the hyperspectral images of 780 *nm*; g, h, and i were the hyperspectral images of 960 *nm*.


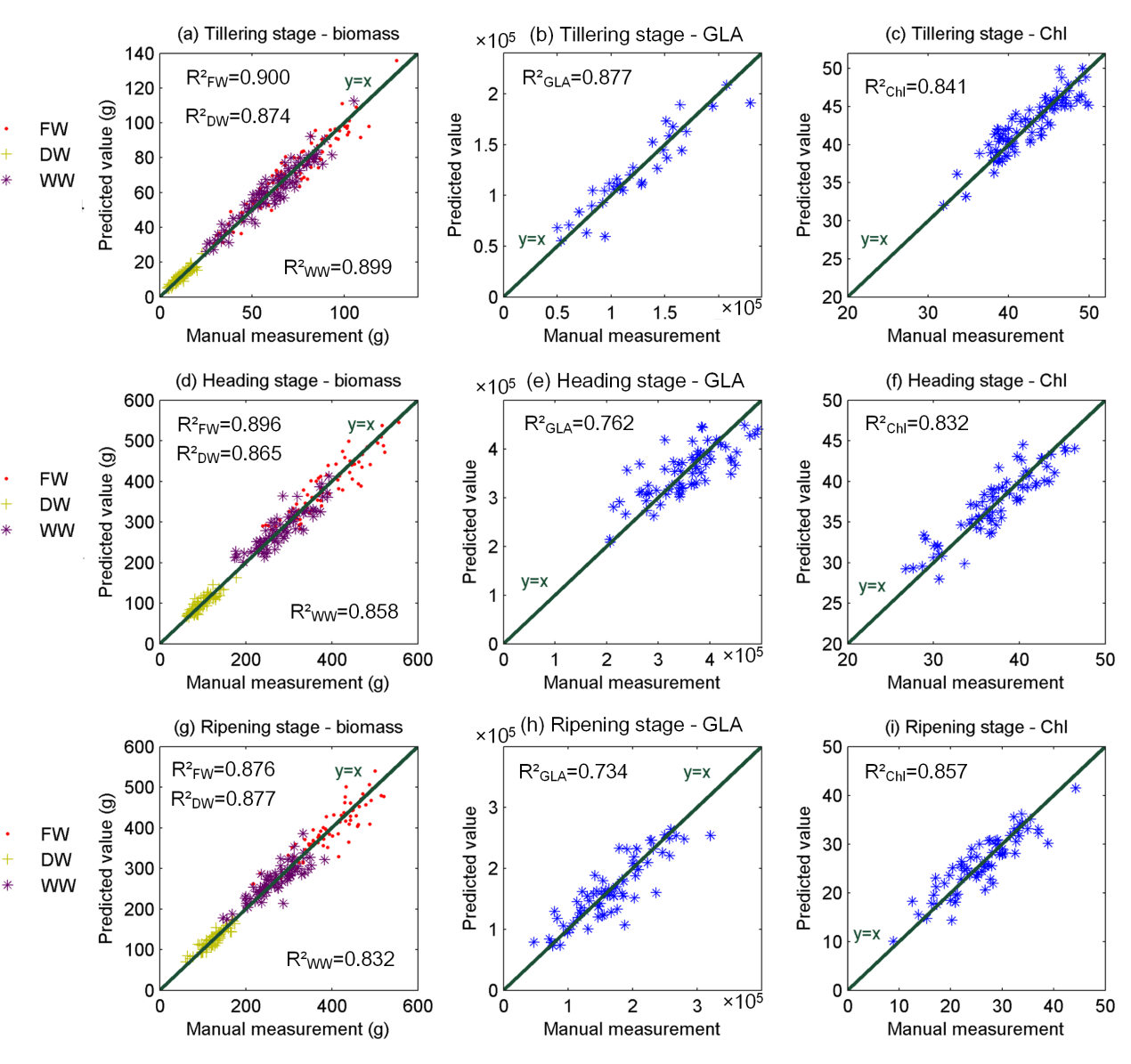


**Supplementary Figure 3 Scatter plots of manual versus automatic measurements of all the phenotypic traits at tillering stage (a-c), heading stage (d-f), and ripening stage (g-i).**

**
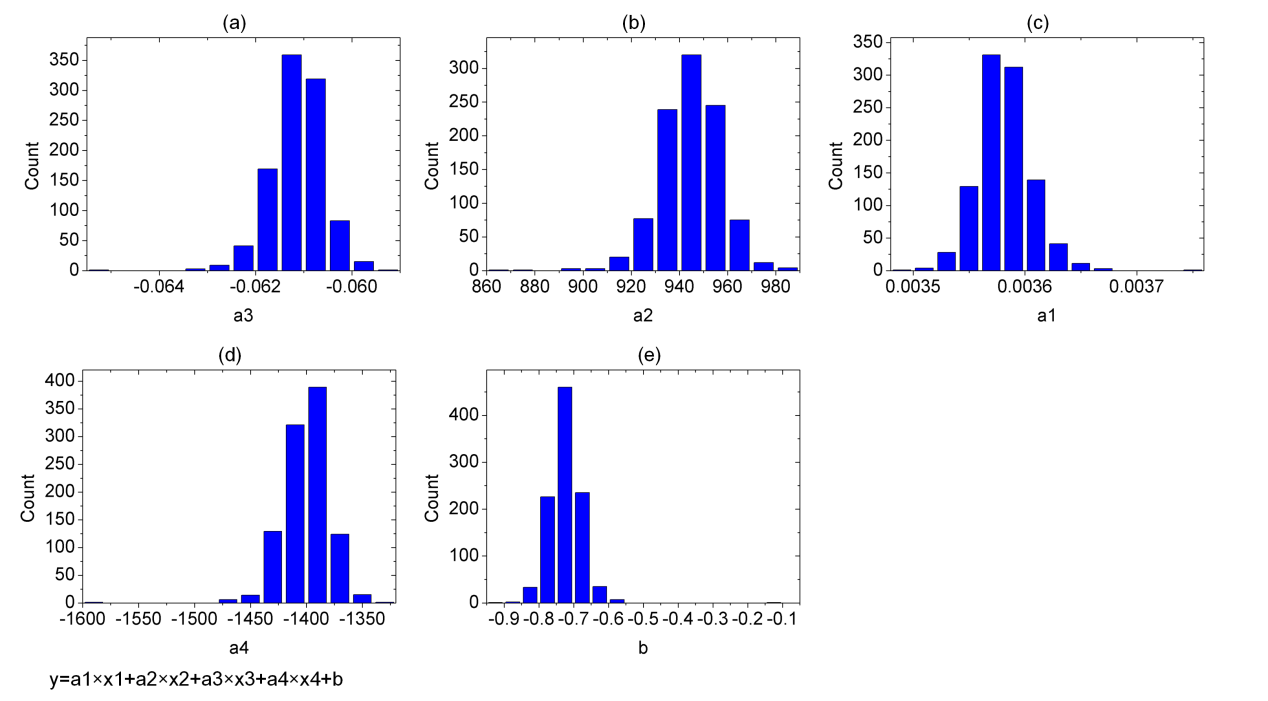
**

**Supplementary Figure 4 The results of the repeatability test of coefficients and constant for the DW modeling at the tillering stage.** The result with 5-fold cross-validation (randomly grouping only once) had lots of randomness. So the 5-fold validation was repeated with 1000 times in this study. It can be seen that the graphics were approximately followed the Gaussian distribution (More than thirty percent of the data distributed on the peak, and eighty percent of the data distributed on the most concentrated areas of the graphic), which indicated the stability of the model.

**
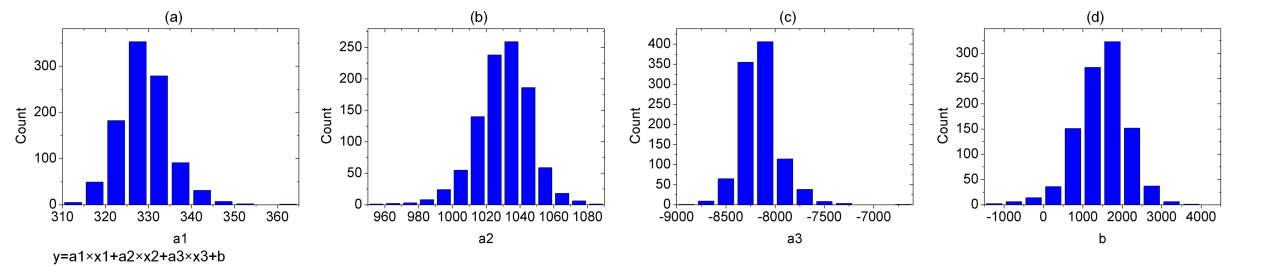
**

**Supplementary Figure 5 The results of the repeatability test of coefficients and constant for the GLA modeling at the tillering stage.** It can be seen that the graphics were approximately followed the Gaussian distribution, which indicated the stability of the model.

**
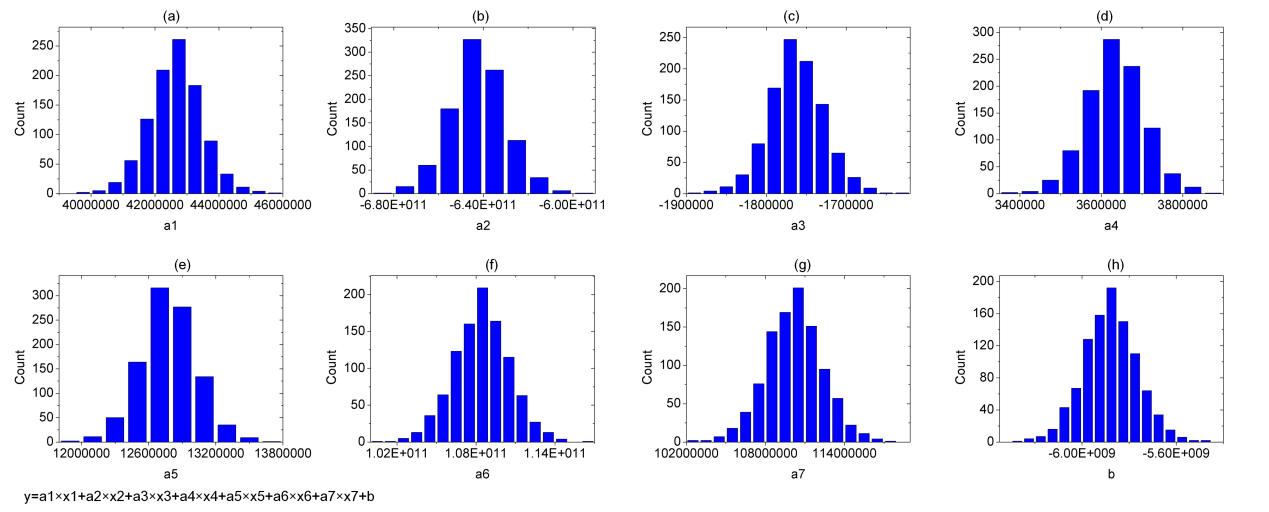
**

**Supplementary Figure 6 The results of the repeatability test of coefficients and constant for the Chl modeling at the tillering stage.** It can be seen that the graphics were approximately followed the Gaussian distribution, which indicated the stability of the model.

**
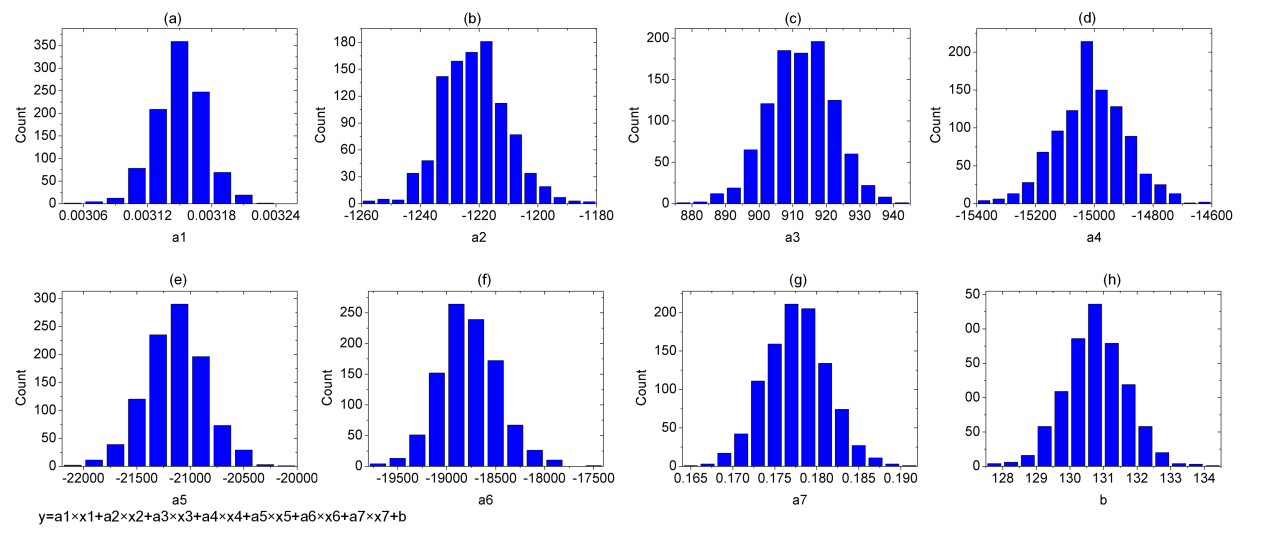
**

**Supplementary Figure 7 The results of the repeatability test of coefficients and constant for the DW modeling at the heading stage.** It can be seen that the graphics were approximately followed the Gaussian distribution, which indicated the stability of the model.

**
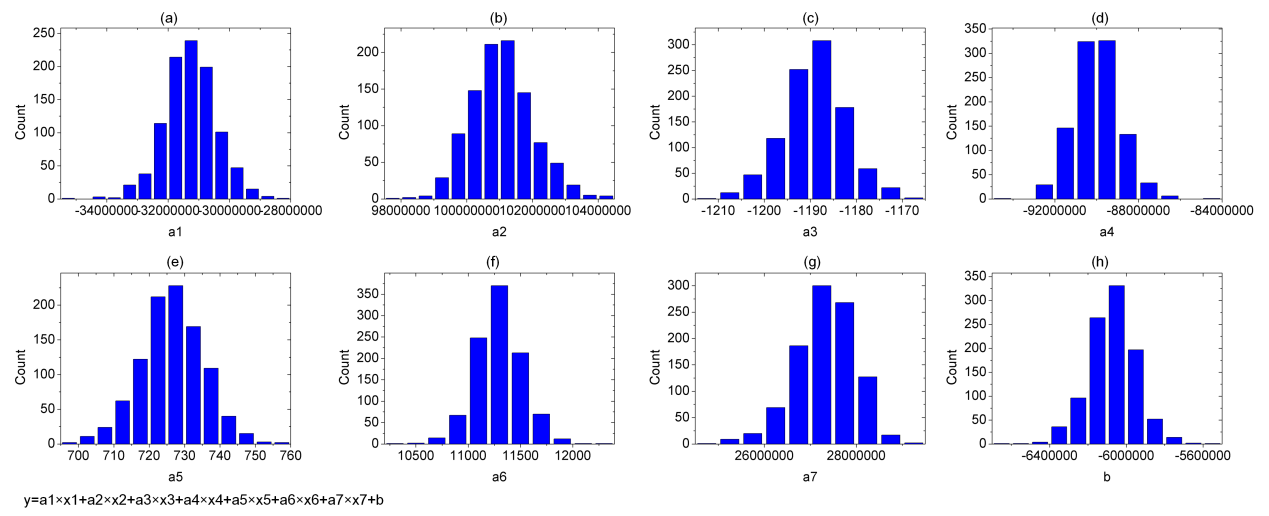
**

**Supplementary Figure 8 The results of the repeatability test of coefficients and constant for the GLA modeling at the heading stage.** It can be seen that the graphics were approximately followed the Gaussian distribution, which indicated the stability of the model.

**
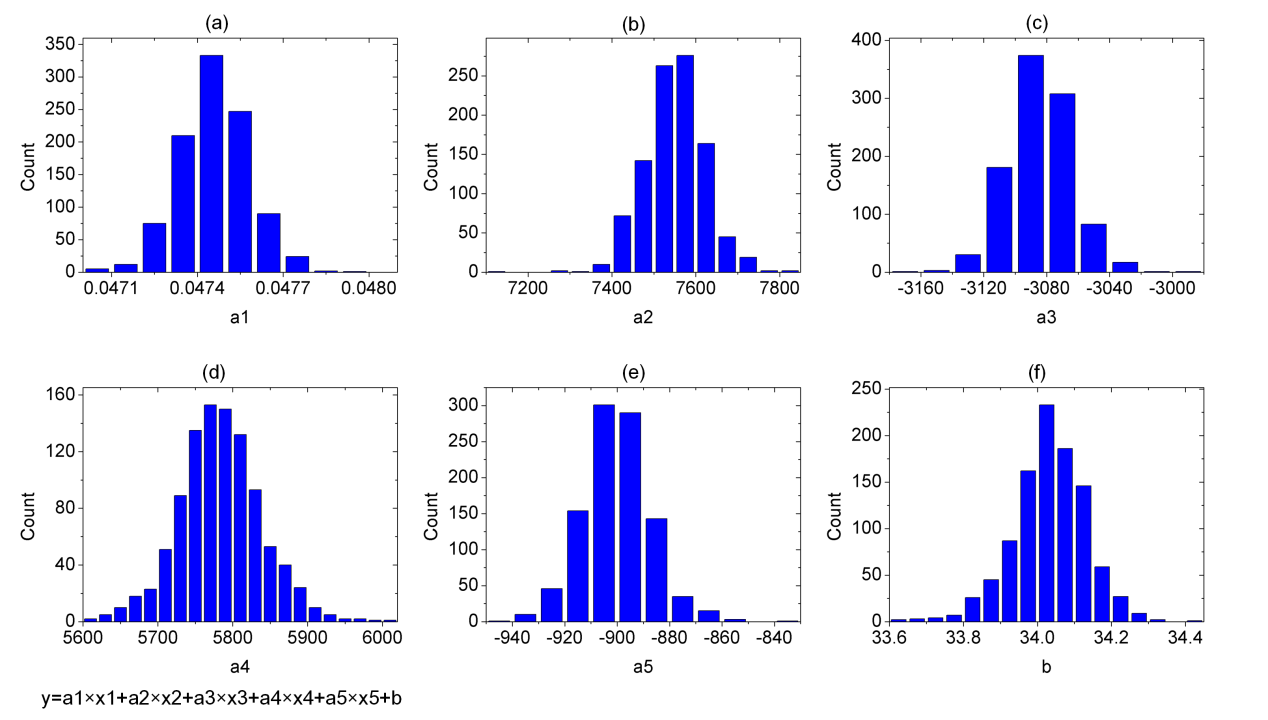
**

**Supplementary Figure 9 The results of the repeatability test of coefficients and constant for the Chl modeling at the heading stage.** It can be seen that the graphics were approximately followed the Gaussian distribution, which indicated the stability of the model.

**
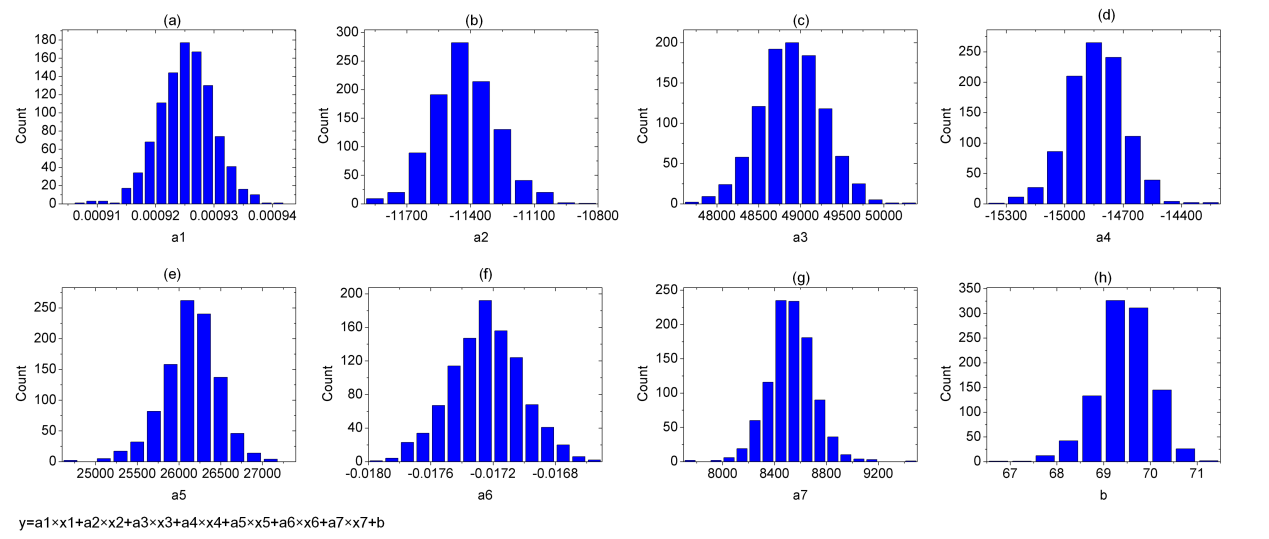
**

**Supplementary Figure 10 The results of the repeatability test of coefficients and constant for the DW modeling at the ripening stage.** It can be seen that the graphics were approximately followed the Gaussian distribution, which indicated the stability of the model.

**
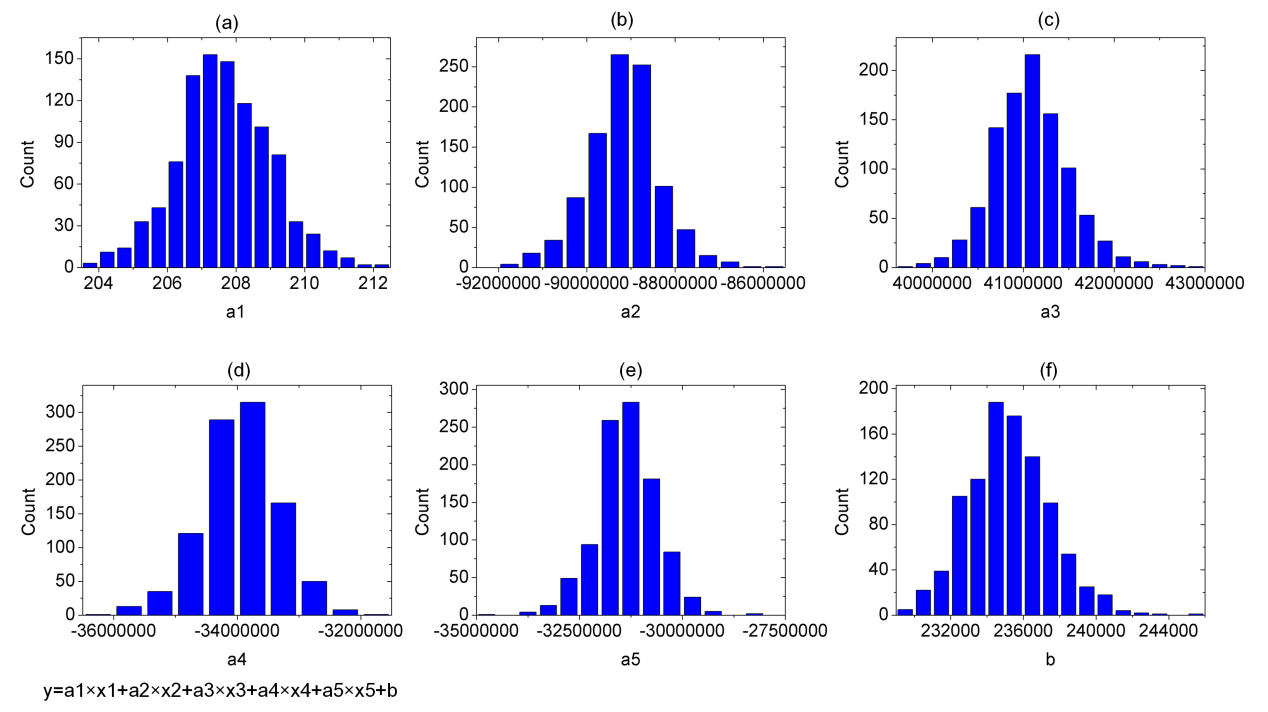
**

**Supplementary Figure 11 The results of the repeatability test of coefficients and constant for the GLA modeling at the ripening stage.** It can be seen that the graphics were approximately followed the Gaussian distribution, which indicated the stability of the model.

**
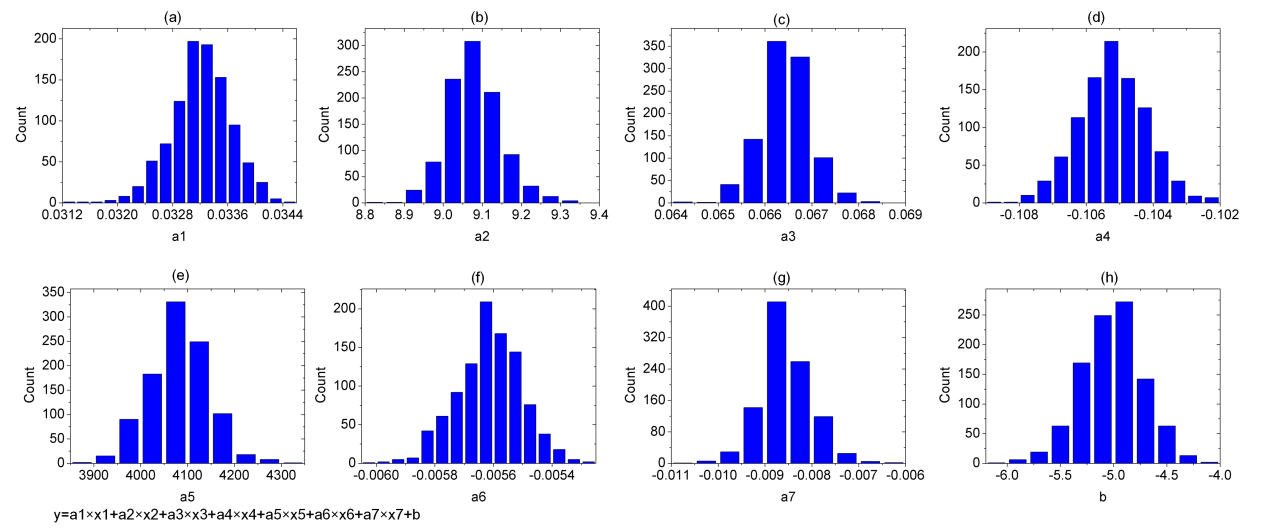
**

**Supplementary Figure 12 The results of the repeatability test of coefficients and constant for the Chl modeling at the ripening stage.** It can be seen that the graphics were approximately followed the Gaussian distribution, which indicated the stability of the model.


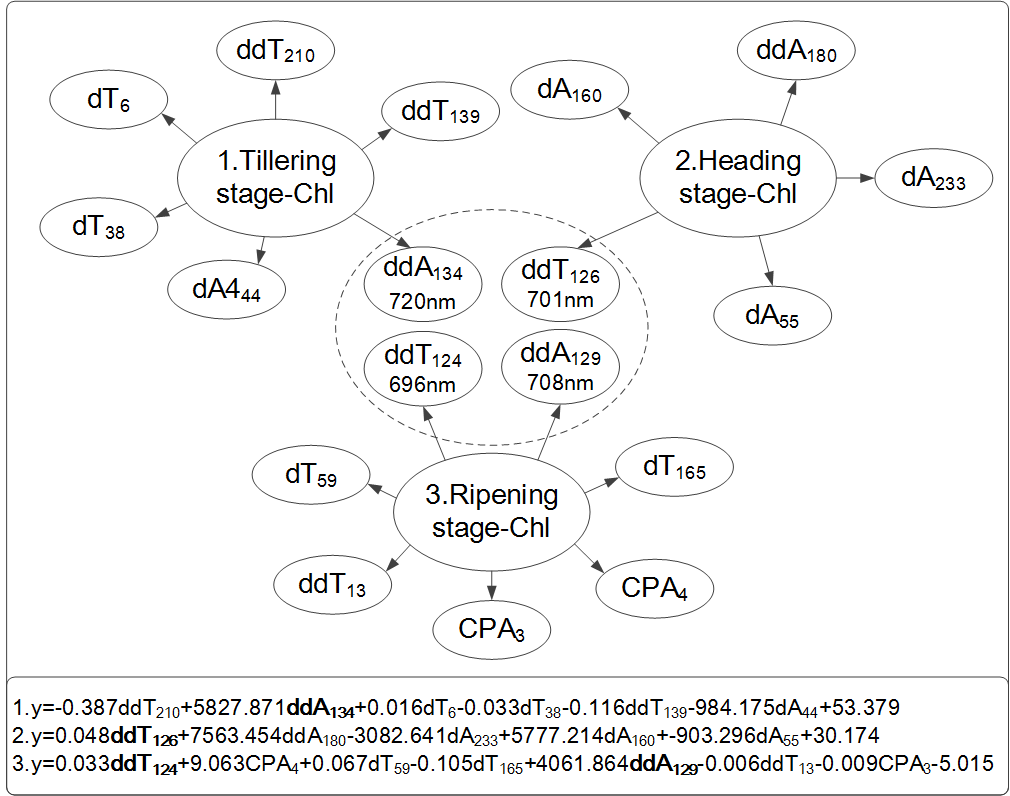


**Supplementary Figure 13 Important hyperspectral indices of the models for the Chl for the three** **growth stages**. The dotted circle represents these wavelengths are close to each other.


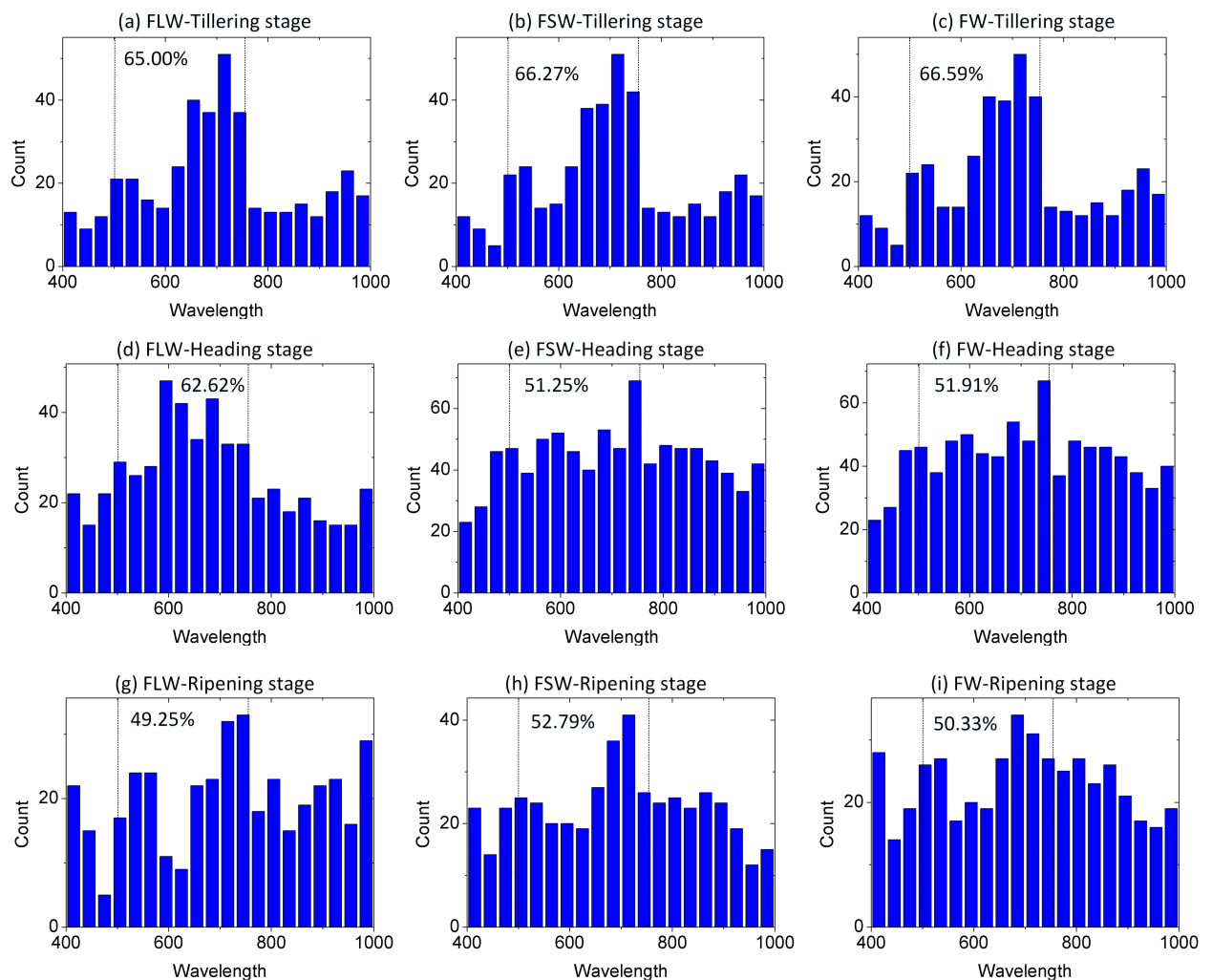


**Supplementary Figure 14 The wavelength frequency distributions of the hyperspectral indices for which the correlation coefficients with FLW, FSW, and FW were greater than 0.3 for the three growth stages.** From the results it can be seen that the wavelengths mainly falls in the region of 500-760 *nm*.


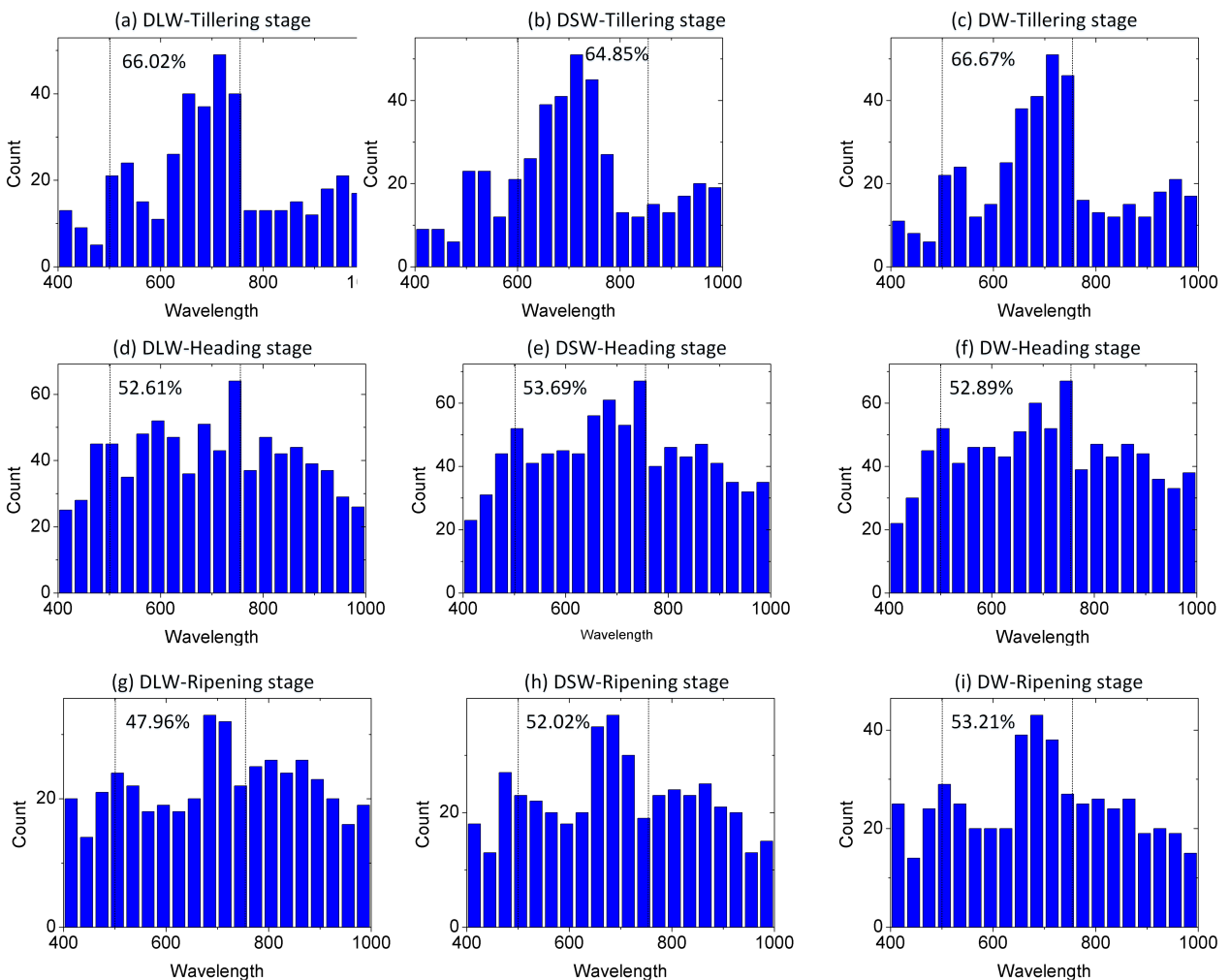


**Supplementary Figure 15 The wavelength frequency distributions of the hyperspectral indices for which the correlation coefficients with DLW, DSW, and DW were greater than 0.3 for the three growth stages.** From the results it can be seen that the wavelengths mainly falls in the region of 500-760 *nm*.


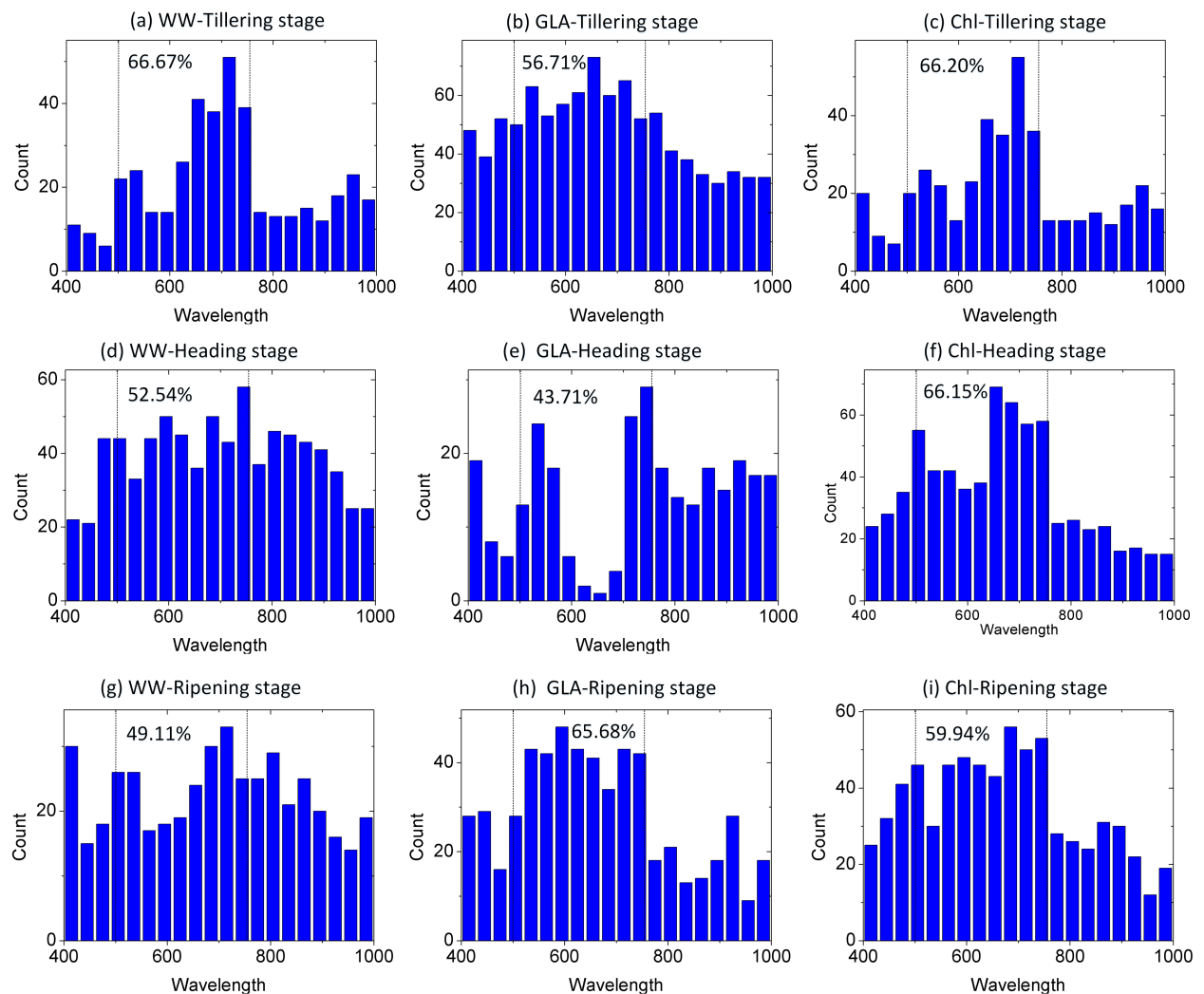


**Supplementary Figure 16 The wavelength frequency distributions of the hyperspectral indices for which the correlation coefficients with WW, GLA, and Chl were greater than 0.3 for the three growth stages.** From the results it can be seen that the wavelengths mainly falls in the region of 500-760 *nm*.


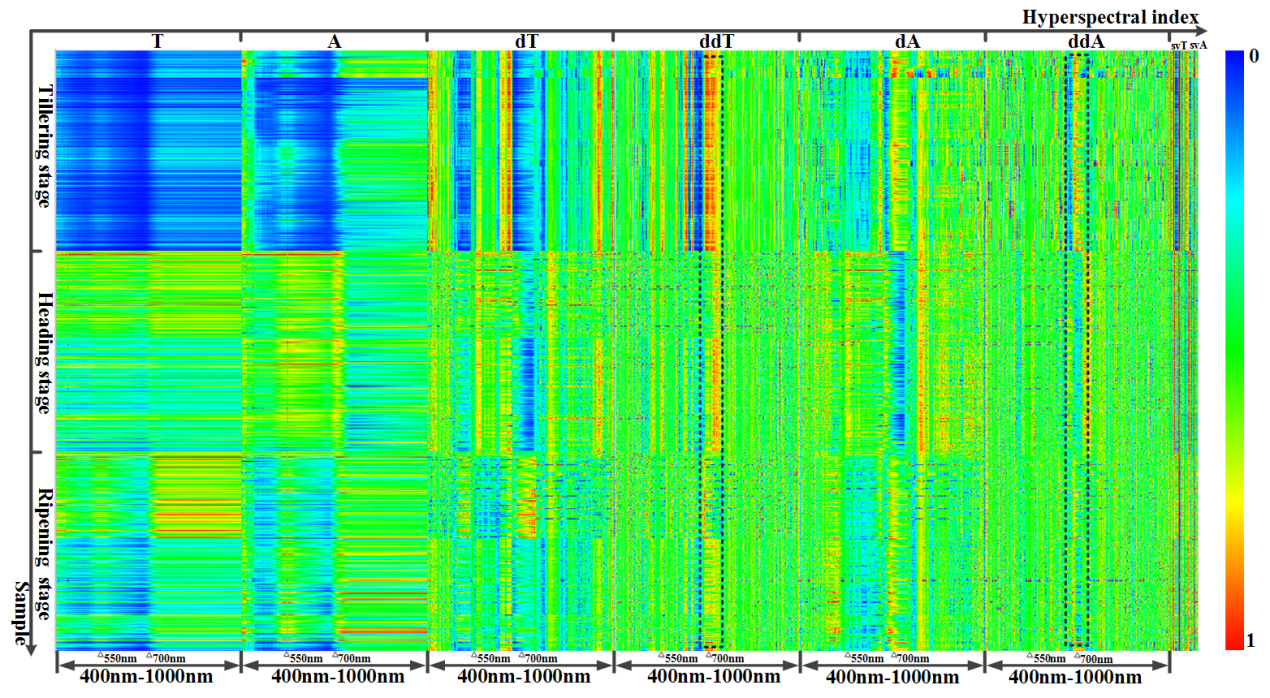


**Supplementary Figure 17 The uniformization of all the original hyperspectral indices of nondestructive samples for the three growth stages.** The hyperspectral indices of the nondestructive samples can be illustrated in a two-dimensional array. The number of samples and the hyperspectral indices at each stages were 270 and 1540, respectively. So the dimension of the array was 810 (=270×3) ×1540. The *x*-axis represented the different types of hyperspectral indices, and the *y*-axis represented the samples during the three stages. For each column, the uniformization was established using the following equation

$$r_{ij}=\frac{R_{ij}-R_{j-min}}{R_{j-max}-R_{j-min}}$$

where *r_ij_* represented the normalized hyperspectral indices of the *i* line and *j* column, *R_ij_* represented the original hyperspectral indices of the *i* line and *j* column, *R_j-min_* represented the minimum value of the hyperspectral indices for the j column, *R_j-max_* represented maximum value of the hyperspectral indices for the j column. (The dotted region represents the important area for the Chl).


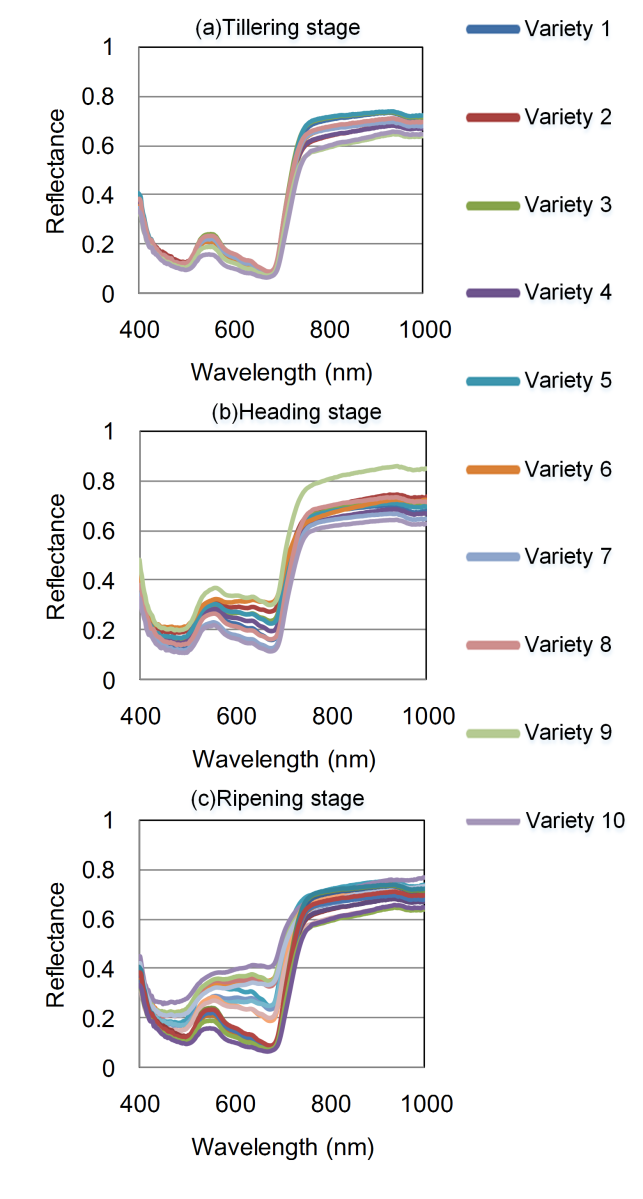


**Supplementary Figure 18 The average reflectance for ten varieties at the three growth stages.** The varieties were randomly selected from all the samples.


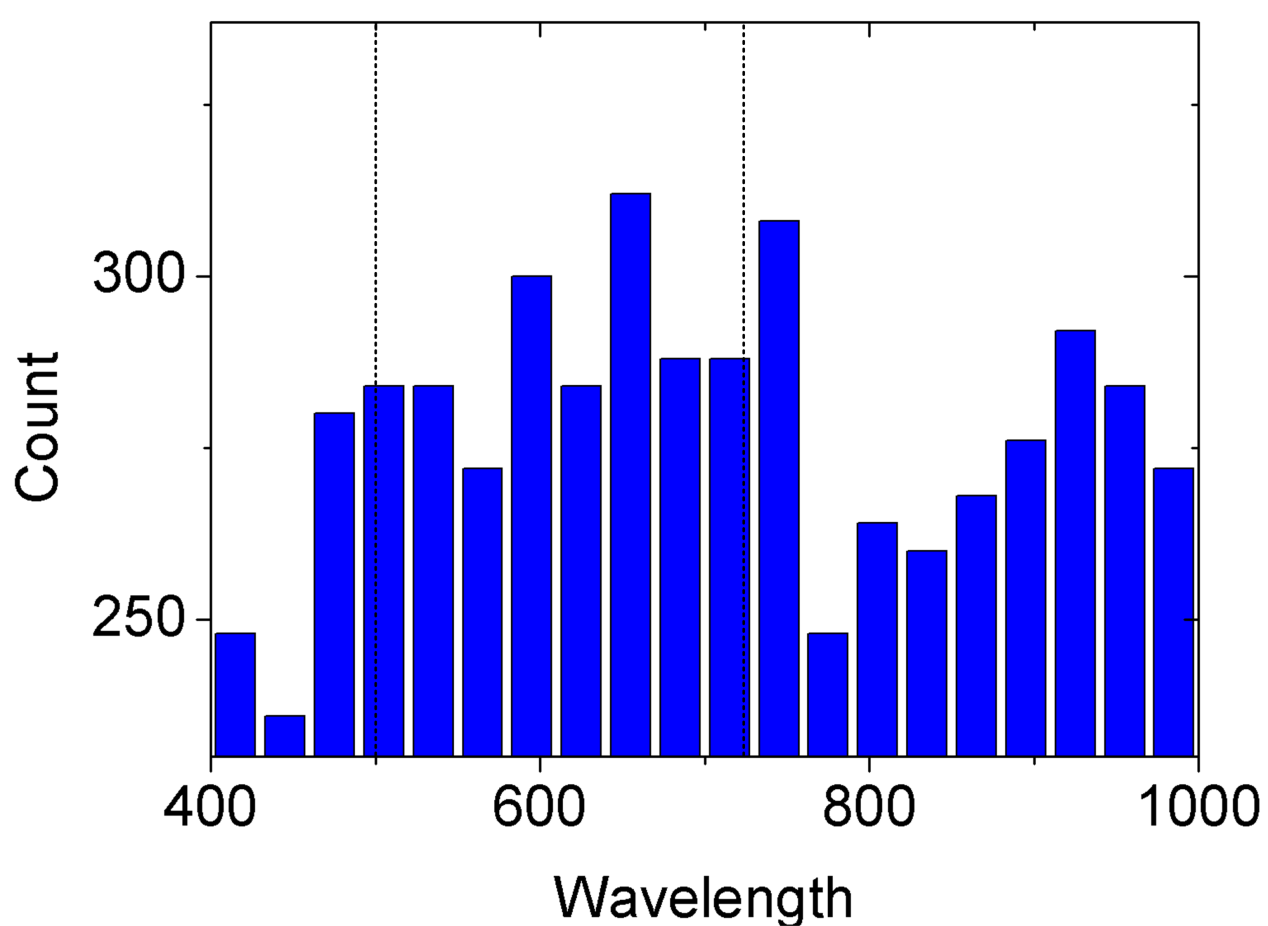


**Supplementary Figure 19 The important wavelengths for classifying the three growth stages**. In order to analyze the difference between the tillering, heading, and ripening stage, single factor analysis of variance for 3 sets (tillering, heading, and ripening stage) of data was used. The confidence interval was 99%. If the *sig* of hyperspectral indices was less than 0.01, the differences between the hyperspectral indices of tillering, heading, and ripening stage were statistically significant. After all of the hyperspectral indices were tested, these hyperspectral indices which had significant differences were chose and the corresponding wavelengths were count. It can be seen that the frequency difference mainly concentrated in the region of 500-760 *nm*.


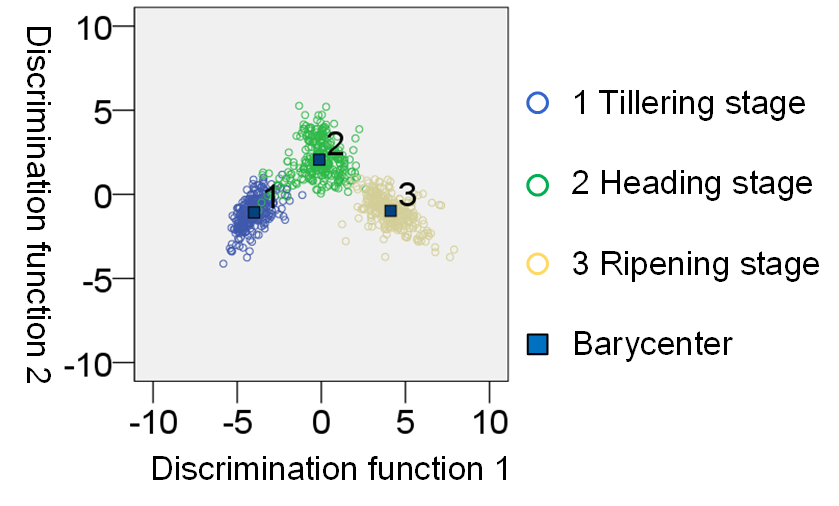


**Supplementary Figure 20 The classified result for the growth stages with hyperspectral indices that were chosen from Supplementary Fig. 19 using stepwise discriminant analysis.** The hyperspectral indices that were chosen from **Supplementary Fig. 19** were selected to perform stepwise discriminant analysis. The correct rate of grouping for the modeling set and cross validation set were 97.7% and 97.3%, respectively. The selected hyperspectral indices were ddT_13_, T_19_, dT_22_, A_36_, A_41_, A_42_, **T_46_, A_47_, ddT_54_, ddA_55_, T_57_, ddT_67_, A_73_, dA_79_, A_81_, dA_81_, T_85_, ddA_85_, ddT_123_, dT_125_, and ddT_127_** (The black font parts were in the region of 500-760 *nm*).


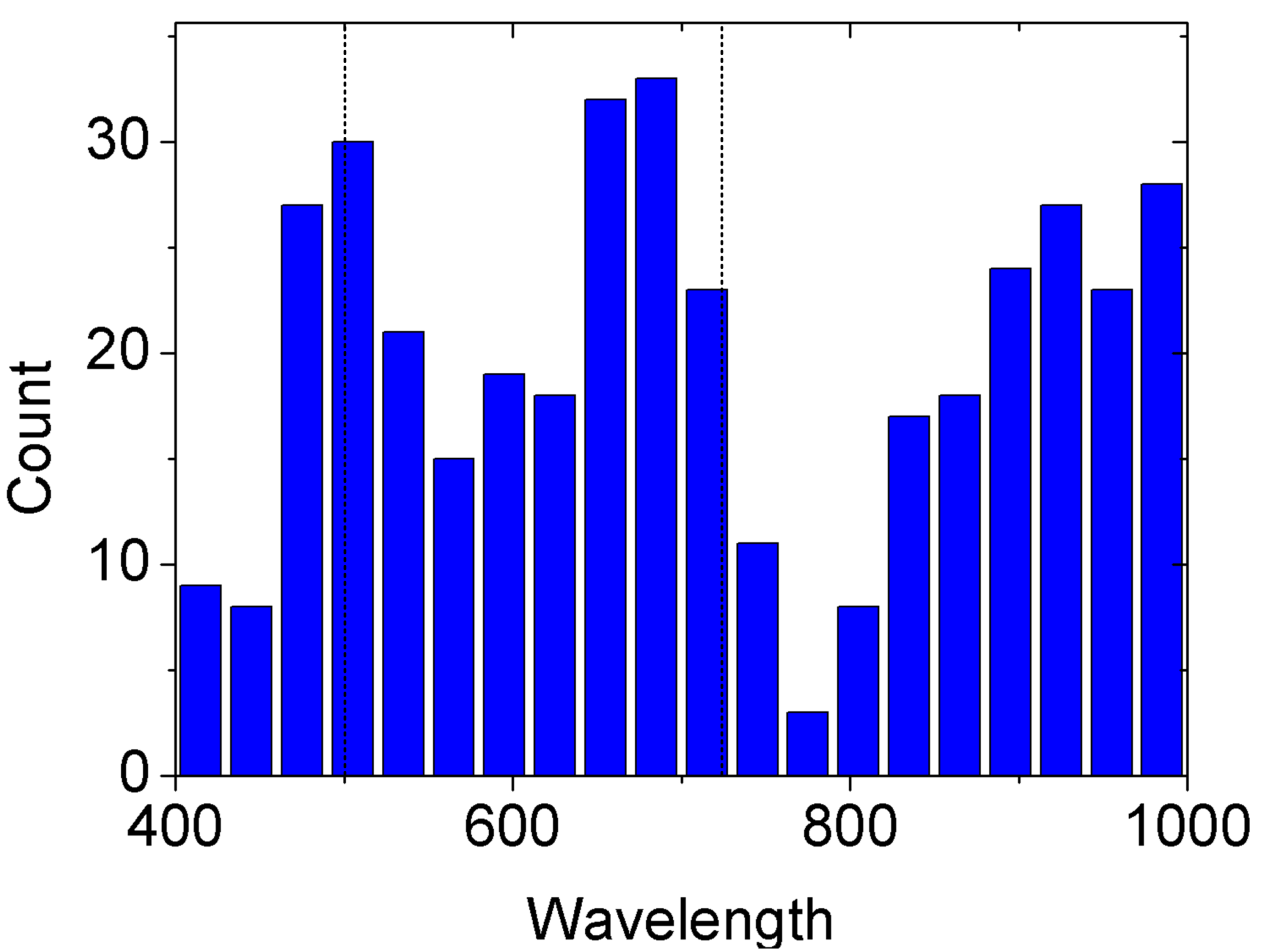


**Supplementary Figure 21 The important wavelengths for classifying the subspecies.** Independent *t*-test was used to detect the difference between the hyperspectral indices of Indica and Japonica rice groups. The confidence interval was 99%. If the *sig* of hyperspectral indices was less than 0.01, the difference between this hyperspectral index of Indica and Japonica group was statistically significant. After all of the hyperspectral indices were tested, these hyperspectral indices which had significant differences were chosen and the corresponding wavelengths were counted. It can be seen that the frequency difference mainly concentrated in the region of 500-760 *nm*.


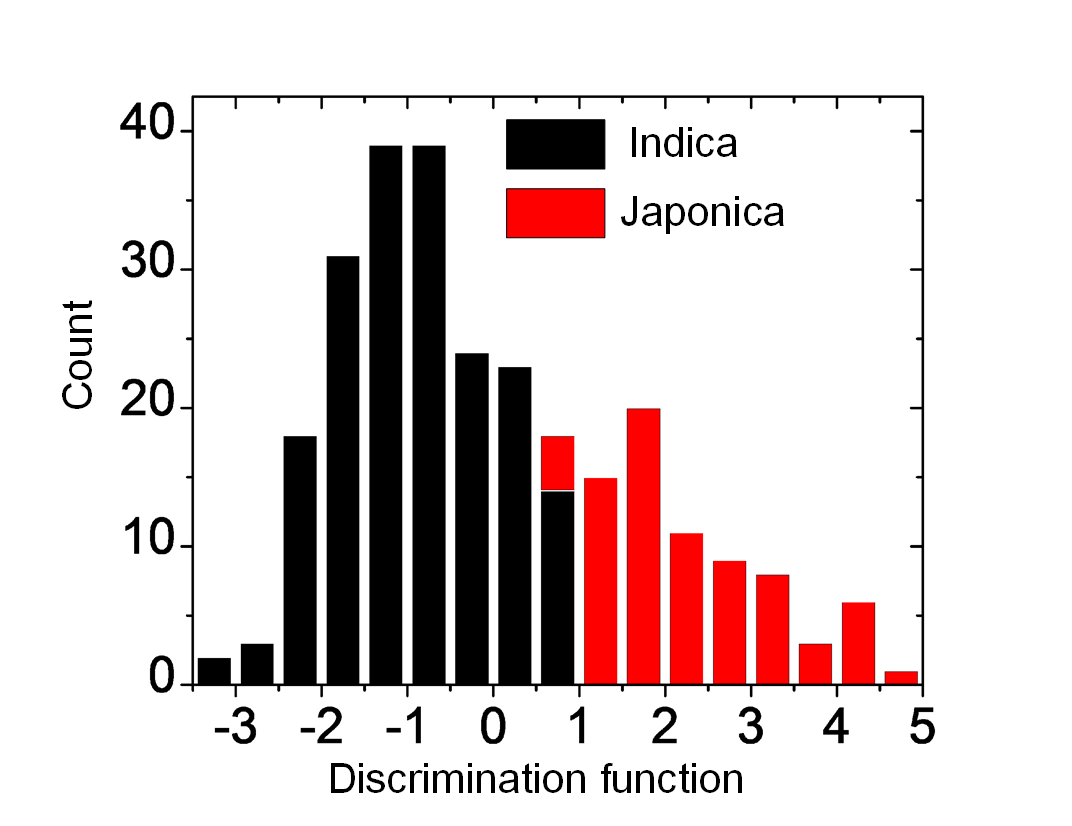


**Supplementary Figure 22 The classified result for subspecies with hyperspectral indices that were chosen from Supplementary Fig. 21 using stepwise discriminant analysis.** The hyperspectral indices chosen from **Supplementary Fig. 21** were used to perform stepwise discriminant analysis. The correct rate of grouping of the Indica and Japonica rice for the modeling set and cross validation set were 95.6% and 93.2%, respectively. The selected hyperspectral indices were dT_29_, ddT_29_, **ddT_165_**, **ddT_234_, dA_115_, dA_159_, dA_185_,** CPT_15_, A_30_, A_34_, **dT_222_, dA_44_, dA_55_, dA_100_, dA_222_, ddA_103_, ddA_247_, CPT_5_, CPT12, ddT_114_, ddA_140_** (The indices in black font are in the region of 500-760 *nm*).


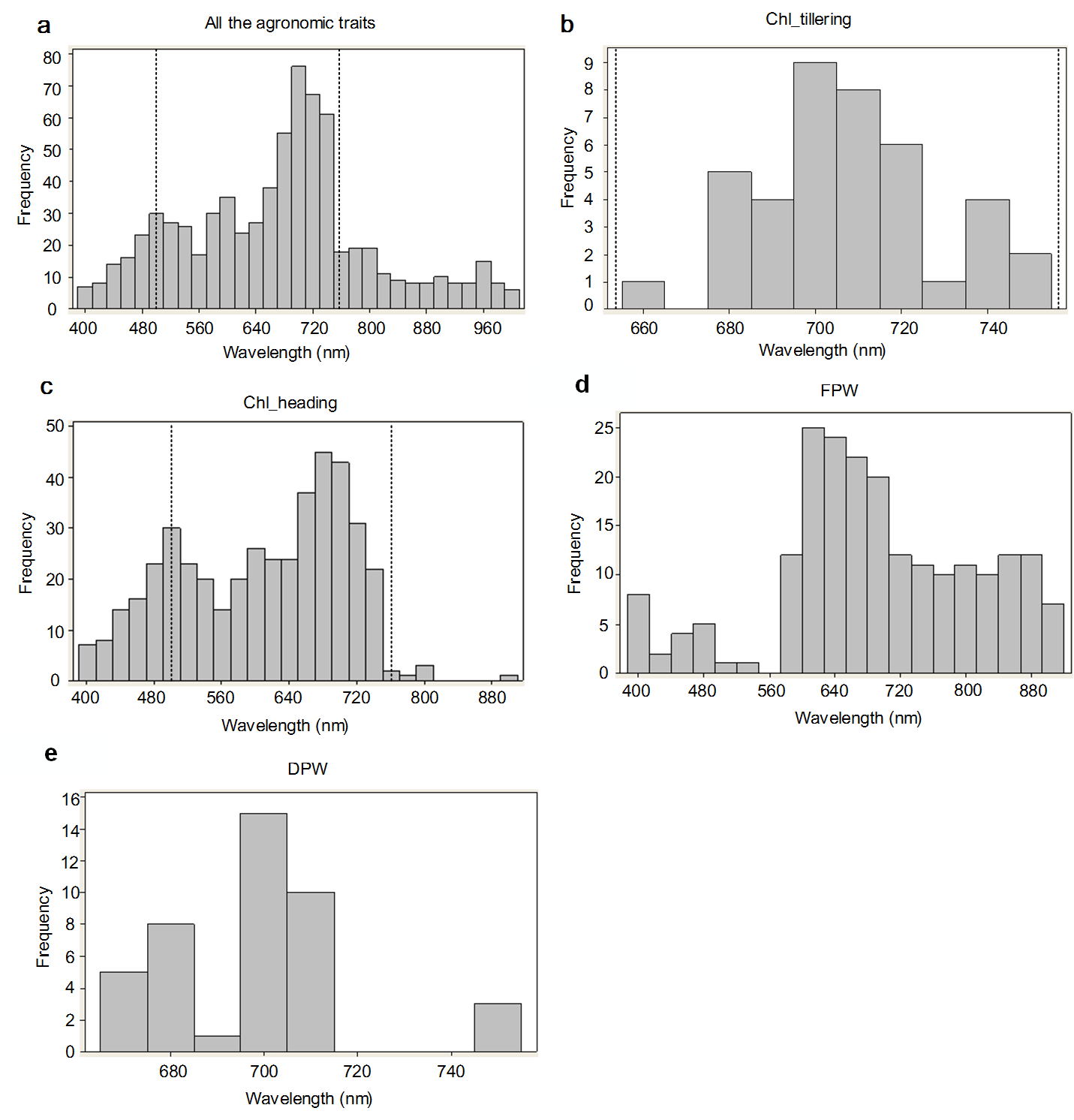


**Supplementary Figure 23 The wavelength distributions of hyper-traits**. The distribution of hyper-traits related to all the agronomic traits (a), the Chl at the tillering stage (b), the heading stage (c), and fresh panicle weight (d), dry panicle weight (e), respectively.


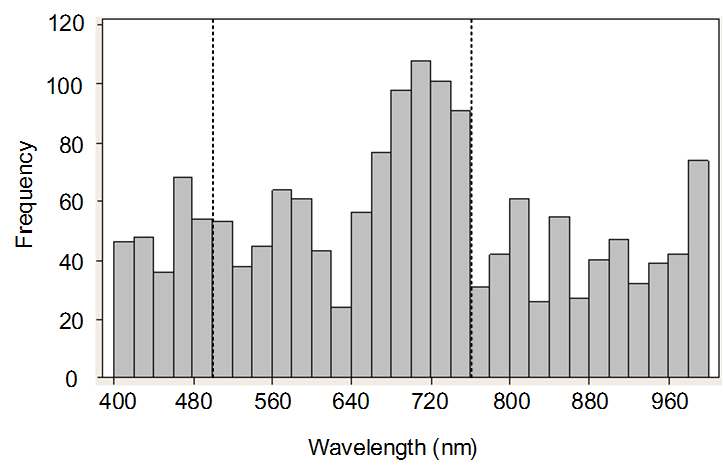


**Supplementary Figure 24 The wavelength distribution of hyperspectral indices co-localized with agronomic traits.**


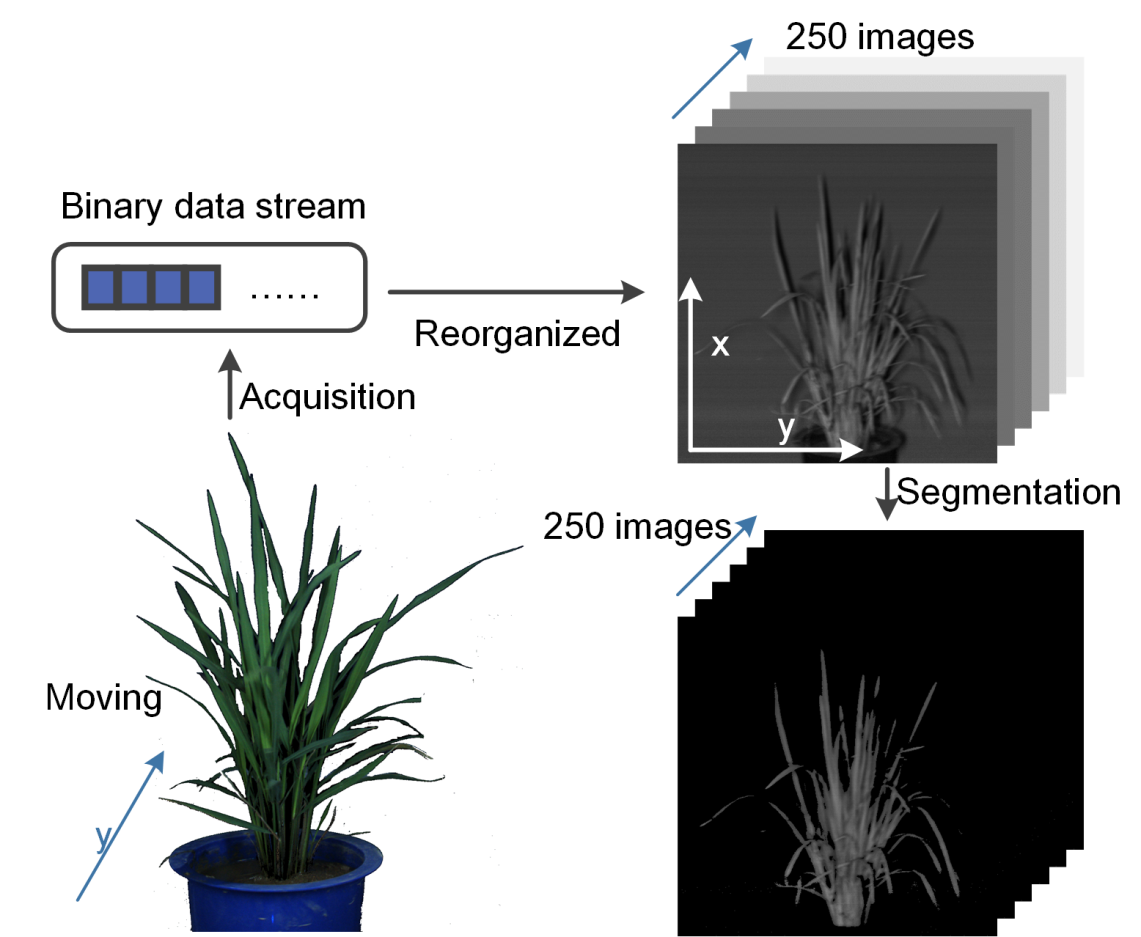


**Supplementary Figure 25 Hyperspectral data analysis pipeline**. With translation stage carrying the rice plant and moving with a constant speed, the hyperspectral camera acquired data continuously. The original acquisition data was binary data stream. After acquisition of one rice plant was finished, the binary data stream was reorganized to generate hyperspectral images of 250 different wavelengths. At last, several image processing steps, including image segmentation, image masking, and data analysis, were performed to extract hyperspectral indices.

**Supplementary Table 1 Hyperspectral indices used in this study (1≤i≤250)**.

| Hyperspectral indices | Definitions |
| --- | --- |
| S | The average area of the plant region of interest (ROI) |
| T_i_ , A_i_ | The total reflectance and average reflectance of the plant ROI, respectively |
| dT_i_, dA_i_ | The first derivative of the *T_i_* and *A_i_*, respectively |
| ddT_i_, ddA_i_ | The second derivative of the *T_i_* and *A_i_*, respectively |
| CPT_1_, CPA_1_ | The normalized ratio of the *CPT_14_* and *CPT_17_*, *CPA_14_* and *CPA_17_*, respectively |
| CPT_2_, CPA_2_ | The normalized ratio of the *CPT_14_* and *CPT_20_*, *CPA_14_* and *CPA_20_*, respectively |
| CPT_3_, CPA_3_ | The ratio of the *CPT_14_* and *CPT_17_*, *CPA_14_* and *CPA_17_*, respectively |
| CPT_4_, CPA_4_ | The ratio of the*CPT_14_* and *CPT_20_*, *CPA_14_* and *CPA_20_*, respectively |
| CPT_5_, CPA_5_ | The normalized ratio of the *CPT_10_* and *CPT_8_*, *CPA_10_* and *CPA_8_*, respectively |
| CPT_6_, CPA_6_ | The ratio of the *CPT_10_* and *CPT_8_*, *CPA_10_* and *CPA_8_*, respectively |
| CPT_7_, CPA_7_ | The red valley position of the *T_i_* and *A_i_*, respectively |
| CPT_8_, CPA_8_ | The red valley reflectance of the *T_i_* and *A_i_*, respectively |
| CPT_9_, CPA_9_ | The green peak position of the *T_i_* and *A_i_*, respectively |
| CPT_10_, CPA_10_ | The green peak reflectance of the *T_i_* and *A_i_*, respectively |
| CPT_11_, CPA_11_ | The green peak area of the *T_i_* and *A_i_*, respectively |
| CPT_12_, CPA_12_ | The red edge position of the *T_i_* and *A_i_*, respectively |
| CPT_13_, CPA_13_ | The red edge amplitude of the *T_i_* and *A_i_*, respectively |
| CPT_14_, CPA_14_ | The red edge area of the *T_i_* and *A_i_*, respectively |
| CPT_15_, CPA_15_ | The yellow edge position of the *T_i_* and *A_i_*, respectively |
| CPT_16_, CPA_16_ | The yellow edge amplitude of the *T_i_* and *A_i_*, respectively |
| CPT_17_, CPA_17_ | The yellow edge area of the *T_i_* and *A_i_*, respectively |
| CPT_18_, CPA_18_ | The blue edge position of the *T_i_* and *A_i_*, respectively |
| CPT_19_ , CPA_19_ | The blue edge amplitude of the *T_i_* and *A_i_*, respectively |
| CPT_20_, CPA_20_ | The blue edge area of the *T_i_* and *A_i_*, respectively |

where *i* indicates the subscript number of the hyperspectral indices, i=1, 2, 3, …, 250. And the relationship between i and the wavelength is shown as the following equation. There were total 1541 hyperspectral indices for one rice plants.

$$Wavelength (nm)=2.4\times i+397.9$$

**Supplementary Table 2 The abbreviation of conventional phenotypic traits in this study*.**

| Full name | Abbreviation | Notes |
| --- | --- | --- |
| Fresh leaf weight | FLW |  |
| Fresh stem weight | FSW |  |
| Fresh panicle weight | FPW | Only measured at the ripening stage |
| Fresh weight | FW | FW=FLW+FSW+FPW |
| Dry leaf weight | DLW |  |
| Dry stem weight | DSW |  |
| Dry panicle weight | DPW | Only measured at the ripening stage |
| Dry weight | DW | DW=DLW+DSW+DPW |
| Water weight | WW | WW=FW-DW |
| Green leaf area | GLA |  |
| Chlorophyll content | Chl |  |

**Supplementary Table 3 The statistic details of the 11 conventional phenotypic traits.**

| Stage | Variable | Standard deviation (SD) | Average value (AV) | Coefficient variation (CV) |
| --- | --- | --- | --- | --- |
| The tillering stage | FLW | 5.38 | 16.90*g* | 27.44% |
|  | FSW | 16.10 | 52.80 *g* | 30.50% |
|  | FW | 21.25 | 72.40 *g* | 29.35% |
|  | DLW | 1.61 | 5.47 *g* | 29.47% |
|  | DSW | 2.41 | 6.23 *g* | 38.74% |
|  | DW | 3.98 | 11.70 *g* | 34.02% |
|  | WW | 17.38 | 60.70 *g* | 28.63% |
|  | GLA | 43135.58 | 122722.10 | 35.15% |
|  | Chl | 3.92 | 42.77 | 9.17% |
| The heading stage | FLW | 10.87 | 62.75 *g* | 17.32% |
|  | FSW | 70.79 | 310.73 *g* | 22.78% |
|  | FW | 75.07 | 381.20 *g* | 19.69% |
|  | DLW | 5.20 | 23.00 *g* | 22.62% |
|  | DSW | 19.60 | 76.41 *g* | 25.65% |
|  | DW | 23.84 | 99.41 *g* | 23.98% |
|  | WW | 55.18 | 281.78 *g* | 19.58% |
|  | GLA | 67617.20 | 351968.80 | 19.21% |
|  | Chl | 4.56 | 37.11 | 12.28% |
| The ripening stage | FLW | 9.15 | 41.90 *g* | 21.83% |
|  | FSW | 62.32 | 298.10 *g* | 20.91% |
|  | FPW | 17.51 | 53.91 *g* | 32.48% |
|  | FW | 69.28 | 393.91 *g* | 17.59% |
|  | DLW | 5.03 | 20.21 *g* | 24.88% |
|  | DSW | 21.70 | 72.37 *g* | 29.99% |
|  | DPW | 11.10 | 30.59 *g* | 36.29% |
|  | DW | 24.16 | 123.18 *g* | 19.62% |
|  | WW | 49.09 | 270.73 *g* | 18.13% |
|  | GLA | 62437.26 | 162599.00 | 38.40% |
|  | Chl | 6.69 | 26.14 | 25.61% |

**Supplementary Table 4 The correlation coefficient between these phenotypic traits***.

| Correlation coefficient | Stage | FLW | FSW | FPW | FW | DLW | DSW | DPW | DW | WW | GLA | Chl |
| --- | --- | --- | --- | --- | --- | --- | --- | --- | --- | --- | --- | --- |
| FLW | Tillering  Heading  Ripening | 1  1  1 |  |  |  |  |  |  |  |  |  |  |
| FSW | Tillering  Heading  Ripening | 0.942  0.725  0.647 | 1  1  1 |  |  |  |  |  |  |  |  |  |
| FPW | Tillering  Heading  Ripening | --  --  -0.042 | --  --  -0.091 | --  --  1 |  |  |  |  |  |  |  |  |
| FW | Tillering  Heading  Ripening | 0.967  0.783  0.703 | 0.996  0.992  0.962 | --  --  0.165 | 1  1  1 |  |  |  |  |  |  |  |
| DLW | Tillering  Heading  Ripening | 0.976  0.861  0.800 | 0.952  0.901  0.854 | --  --  -0.084 | 0.968  0.914  0.853 | 1  1  1 |  |  |  |  |  |  |
| DSW | Tillering  Heading  Ripening | 0.918  0.481  0.597 | 0.968  0.848  0.852 | --  --  0.307 | 0.966  0.831  0.767 | 0.954  0.769  0.754 | 1  1  1 |  |  |  |  |  |
| DPW | Tillering  Heading  Ripening | --  --  -0.199 | --  --  -0.191 | --  --  0.969 | --  --  --0.047 | --  --  -0.218 | --  --  -0.365 | --  --  1 |  |  |  |  |
| DW | Tillering  Heading  Ripening | 0.952  0.583  0.611 | 0.972  0.894  0.855 | --  --  0.152 | 0.978  0.883  0.888 | 0.983  0.850  0.785 | 0.992  0.990  0.887 | --  --  0.087 | 1  1  1 |  |  |  |
| WW | Tillering  Heading  Ripening | 0.965  0.813  0.692 | 0.996  0.963  0.937 | --  --  0.158 | 0.999  0.979  0.974 | 0.959  0.876  0.817 | 0.954  0.702  0.646 | --  --  0.024 | 0.966  0.769  0.761 | 1  1  1 |  |  |
| GLA | Tillering  Heading  Ripening | 0.944  0.741  0.694 | 0.899  0.368  0.214 | --  --  0.068 | 0.917  0.440  0.301 | 0.942  0.523  0.286 | 0.906  0.216  0.188 | --  --  -0.054 | 0.926  0.291  0.204 | 0.910  0.472  0.324 | 1  1  1 |  |
| Chl | Tillering  Heading  Ripening | -0.744  -0.407  0.124 | -0.708  -0.577  -0.253 | --  --  -0.218 | -0.7025  -0.578  -0.267 | -0.729  -0.623  -0.191 | -0.691  -0.736  -0.170 | --  --  -0.269 | -0.714  -0.741  -0.316 | -0.723  -0.466  -0.221 | -0.744  -0.295  0.349 | 1  1  1 |

**Supplementary Table 5 The results of models** **for the conventional phenotypic traits with the linear stepwise regression (LSR).**

| Stage | Dependent Variable | N | R^2^ | Model | MAPE | SD_APE_ |
| --- | --- | --- | --- | --- | --- | --- |
| The tillering stage | FW | 91 | 0.900 | y=0.041CPT_7_+0.102dT_142_-11557dA_113_-7.940 | 7.77% | 5.35% |
|  | DW | 91 | 0.874 | y=0.004CPT_9_+944.829dA_60_-0.061dT_132_-1390.779dA_169_-0.736 | 9.74% | 8.00% |
|  | WW | 91 | 0.899 | y=0.033CPT_7_+0.085dT_142_-9364.209dA_113_-5.226 | 7.65% | 5.21% |
|  | GLA | 34 | 0.877 | y=326.504dT_53_+1036.148dT_29_-8249.391ddT_97_+1605.137 | 10.49% | 9.07% |
|  | Chl | 91 | 0.841 | y=-0.387ddT_210_+5827.871ddA_134_+0.016dT_6_-0.033dT_38_-0.116ddT_139_-984.175dA_44_+53.379 | 2.97% | 2.32% |
| The heading stage | FW | 71 | 0.896 | y=0.011CPT_13_-6307.386A_33_-54046.4ddA_204_+1966.400A_90_+2124.555A_12_-362317ddA_91_+2.794ddT_91_+321.708 | 5.38% | 3.84% |
|  | DW | 71 | 0.865 | y=0.003T_106_-1224.98A_22_+914.475A_12_-15043.6ddA_124_-21120.6dA_117_-18863.1dA_218_+0.179dT_178_+130.911 | 7.22% | 5.36% |
|  | WW | 71 | 0.858 | y=0.012T_116_-2023.823A_33_-21129.8dA_221_-19530.6ddA_204_-30950.8dA_231_-61.559CPA_15_-0.059ddT_12_+419.446 | 6.14% | 5.18% |
|  | GLA | 71 | 0.762 | y=-3E+007dA_162_+1E+008dA_82_-1188.445dT_70_-9E+007dA_211_+727.883dT_90_+11279.27CPA_11_+3E+007dA_40_+6068561 | 8.03% | 7.13% |
|  | Chl | 71 | 0.832 | y=0.048ddT_126_+7563.454ddA_180_-3082.641dA_233_+5777.214dA_160_+-903.296dA_55_+30.174 | 4.06% | 3.15% |
| The ripening stage | FW | 69 | 0.876 | y=0.003S-69785.2dA_117_+0.539dT_85_-61263.3dA_215_+147760.9ddA_134_+50462.515ddA_106_-0.227dT_232_+399.316 | 4.65% | 3.91% |
|  | DW | 69 | 0.877 | y=9.2E-04S-11419.4dA_116_+49010.493ddA_134_-14813.7dA_234_+26162.314ddA_143_-0.017dT_7_+8500.802ddA_171_+69.67 | 5.44% | 5.43% |
|  | WW | 69 | 0.832 | y=0.002S+17498.862dA_31_+86795.503ddA_134_-41144.9dA_155_-50872.9dA_209_-0.076ddT_20_-67542.5ddT_82_+286.355 | 5.77% | 4.47% |
|  | GLA | 69 | 0.734 | y=202.226dT_136_-9E+07dA_155_+4E+07dA_226_-3E+07ddA_49_-4E+07dA_197_+241777.6 | 13.36% | 12.69% |
|  | Chl | 69 | 0.857 | y=0.033ddT_124_+9.063CPA_4_+0.067dT_59_-0.105dT_165_+4061.864ddA_129_-0.006ddT_13_-0.009CPA_3_-5.015 | 8.18% | 7.55% |

**Supplementary Table 6 The result of 5-fold cross-validation between the conventional phenotypic traits and hyperspectral indices (randomly grouping once)**.

| Stage | Dependent Variable | Training set | | | Testing set | | |
| --- | --- | --- | --- | --- | --- | --- | --- |
|  |  | R² | MAPE | SD_APE_ | R² | MAPE | SD_APE_ |
| The tillering stage | FW | 0.901 | 7.72% | 5.30% | 0.890 | 8.10% | 5.55% |
|  | DW | 0.876 | 9.99% | 8.21% | 0.856 | 10.45% | 8.59% |
|  | WW | 0.900 | 7.59% | 5.19% | 0.888 | 8.22% | 5.62% |
|  | GLA | 0.877 | 10.40% | 8.88% | 0.846 | 11.68% | 8.87% |
|  | Chl | 0.852 | 2.80% | 2.13% | 0.812 | 3.17% | 2.41% |
| The heading stage | FW | 0.901 | 5.16% | 3.64% | 0.846 | 6.63% | 4.50% |
|  | DW | 0.867 | 7.17% | 5.24% | 0.824 | 8.32% | 6.04% |
|  | WW | 0.861 | 5.81% | 5.29% | 0.816 | 6.59% | 6.29% |
|  | GLA | 0.764 | 7.86% | 6.63% | 0.722 | 8.60% | 7.83% |
|  | Chl | 0.833 | 4.06% | 3.08% | 0.800 | 4.47% | 3.35% |
| The ripening stage | FW | 0.877 | 4.56% | 3.83% | 0.832 | 5.46% | 4.56% |
|  | DW | 0.879 | 5.40% | 5.37% | 0.844 | 6.28% | 5.75% |
|  | WW | 0.836 | 5.69% | 4.40% | 0.792 | 6.39% | 5.03% |
|  | GLA | 0.794 | 13.77% | 14.62% | 0.727 | 15.47% | 19.94% |
|  | Chl | 0.859 | 8.08% | 7.31% | 0.820 | 9.27% | 8.36% |

**Supplementary Table 7 The result of principal component analysis (PCA) and linear stepwise regression (LSR) between the destructive phenotypic traits and hyperspectral indices*.**

| Stage |  | Dependent Variable | PCA&LSR | | LSR | |
| --- | --- | --- | --- | --- | --- | --- |
|  |  |  | R^2^ | MAPE | R^2^ | MAPE |
| The tillering stage |  | FW | 0.865 | 8.89% | 0.900 | 7.77% |
|  |  | DW | 0.830 | 10.89% | 0.874 | 9.74% |
|  |  | WW | 0.865 | 8.82% | 0.899 | 7.65% |
|  |  | GLA | 0.843 | 12.18% | 0.877 | 10.49% |
|  |  | Chl | 0.740 | 3.89% | 0.841 | 2.97% |
| The heading stage |  | FW | 0.740 | 8.07% | 0.896 | 5.38% |
|  |  | DW | 0.728 | 10.01% | 0.865 | 7.22% |
|  |  | WW | 0.659 | 9.18% | 0.858 | 6.14% |
|  |  | GLA | 0.429 | 12.36% | 0.762 | 8.03% |
|  |  | Chl | 0.674 | 5.68% | 0.832 | 4.06% |
| The ripening stage |  | FW | 0.674 | 8.34% | 0.876 | 4.65% |
|  |  | DW | 0.667 | 9.87% | 0.877 | 5.44% |
|  |  | WW | 0.608 | 9.20% | 0.832 | 5.77% |
|  |  | GLA | 0.539 | 23.85% | 0.734 | 13.36% |
|  |  | Chl | 0.689 | 12.23% | 0.857 | 8.18% |

***** PCA is the most common method for data dimension reduction. So the PCA method was used to analyze all the hyperspectral indices in this study. And the principal components (PC) with the eigenvalues greater than 1 were selected and forward to the following LSR steps. There were 26, 38, and 40 PCs for the three growth stages, respectively.

**Supplementary Table 8 The results of estimating the biomass, leaf area, and Chl with different spectral resolutions*.**

| Stage | Dependent Variable | R² with different resolution* | |
| --- | --- | --- | --- |
|  |  | 2.4 nm | 4.8 nm |
| The tillering stage | FW | 0.906 | 0.901 |
|  | DW | 0.874 | 0.857 |
|  | WW | 0.908 | 0.901 |
|  | GLA | 0.876 | 0.893 |
|  | Chl | 0.792 | 0.766 |
| The heading stage | FW | 0.839 | 0.841 |
|  | DW | 0.796 | 0.783 |
|  | WW | 0.790 | 0.778 |
|  | GLA | 0.605 | 0.658 |
|  | Chl | 0.808 | 0.773 |
| The ripening stage | FW | 0.797 | 0.823 |
|  | DW | 0.817 | 0.790 |
|  | WW | 0.728 | 0.753 |
|  | GLA | 0.716 | 0.717 |
|  | Chl | 0.787 | 0.733 |

***** In this paper, the hyperspectral imaging system was running with the best spectral resolution 2.4 *nm*, and we also discussed whether the modeling accuracy would be declined if the data was merged with lower spectral resolution. The comparison between 2.4 *nm* and 4.8 *nm* was shown in the table. It can be seen that the results with the resolution of 4.8 *nm* were similar to the results with the resolution of 2.4 *nm*. To provide the high-quality data for GWAS, the hyperspectral indices in this study were still the results with the resolution of 2.4 *nm*. In future, to consider reducing the acquisition time, 4.8 *nm* may be a better choice.

**Supplementary Table 14 The correlation (R^2^) between the GLA and Chl and projected area (S).**

| Stage | GLA vs S | Chl vs S |
| --- | --- | --- |
| The tillering stage | 0.670 | 0.221 |
| The heading stage | 0.197 | 0.358 |
| The ripening stage | 0.031 | 0.214 |

**Supplementary references**

[1] HUANG J, WANG F, WANG X. Hyperspectral experiment for paddy rice remote sensing; Huang JQ, Chen JY, editors. Hangzhou [M]. Zhejiang University Press. 2010.
